# Supplementary material for: Efficient representations of tumor diversity with paired DNA-RNA aberrations
Source: PLoS Comput Biol. 2021 Jun 11;17(6):e1008944. doi: 10.1371/journal.pcbi.1008944 (PMC8221796; doi:10.1371/journal.pcbi.1008944)
Supplement: S1 Text — Specifically, this supporting file contains two subsections. In the Supplementary Table subsection, Tables A—B show results pertaining to the selection of parameters k and L. Tables C—D show the basic statistics of interactions before and after filters. Tables F—U illustrate the statistics of “Optimal Covering” and core set with associated probabilities for 5 tissues at 3 levels. Tables V—I display the divergence probabilities at different cancer subtypes. Tables II and III show the entropy analysis across distinct tissues. In Supplementary Fig subsection, Fig A shows probabilities relating to target cores, and Fig B shows effects of filter thresholds on fraction of samples covered. Figs C—G show pair covering network across different cancers. Figs H—J show annotated network for cancer subtypes. Figs L—M show the complete core set across tissues. Figs N—P display coding trees for cancer subtypes. (PDF) [file pcbi.1008944.s001.pdf]

1 Supplementary Text

1.1 $k = 3$ v.s $k = 4$

Recall from Section 2.2.2, we described the process of building source-target pairs (STPs) using the Reactome pathway database, and we use $k = 3$ in our experiments where k is the length of STP $g_s \Rightarrow g_t$.

Preliminary computations show that with $k > 4$, the number of matched STPs is quite large and provokes computational problems, while with $k = 1$ or $k = 2$, STPs of possible significance may be omitted. Therefore we may consider $k = 3$ and $k = 4$ to be candidates for this hyper-parameter for further consideration.

Proceeding from these initial conclusions, we now offer a more detailed analysis between $k = 3$ and $k = 4$. As seen in Table C, the number of interactions with $k \leq 4$ is about 5 times that with $k \leq 3$. Also, the optimal covering statistics for multiple tissues (e.g., breast, colon, liver, lung) are quite similar at all three levels (i.e., STP, source, target) between Supplementary Table A (below) and Table 2 (main paper). Moreover, for target level covering with $k = 4$, Supplementary Figure A (below) indicates that given a target gene g_t is RNA aberrant, there is strong likelihood that at least one of its sources g_s is DNA aberrant, with $P(\text{upstream DNA}|\text{RNA})$ between $0.7 - 1$ for indicated 4 tissues. For colon cancer and lung cancer at target level, $P(\text{upstream DNA}|\text{RNA})$ is even higher than 0.9 for all core. These relatively high values of $P(\text{upstream DNA}|\text{RNA})$ suggest that we could very likely be able to discover an upstream DNA aberration for any downstream RNA aberration – which may not be reasonable, since a gene can be RNA-aberrant for many reasons other than an upstream genetic aberration (consider for instance, an epigenetic cause or fusion-related). This would suggest $k = 3$ to be the optimal choice providing the most biologically meaningful and reasonable tableau of paired aberrations as input for the minimal covering optimization.

Tissue	Samples	Covering Type	Quantity	Size of solution	Total features in solution	Fraction of samples covered	Size of core set
Breast	953	STP	12890	73	563	0.977	16
		Source	1133	59	95	0.983	42
		Target	285	45	75	0.983	32
Colon	207	STP	4350	7	78	1.000	3
		Source	1138	7	220	1.000	2
		Target	275	8	137	1.000	2
Kidney	336	STP	857	41	113	0.929	24
		Source	232	43	68	0.973	33
		Target	276	53	92	0.991	28
Liver	360	STP	3438	36	167	0.978	11
		Source	866	34	149	0.992	12
		Target	347	32	125	0.983	10
Lung	465	STP	10291	22	87	0.991	6
		Source	1698	23	23	0.996	23
		Target	373	21	75	0.994	5
Prostate	491	STP	1977	64	119	0.827	43
		Source	571	67	115	0.890	49
		Target	236	66	97	0.845	49

Table A: Statistics of optimal coverings with $k = 4$. This table shows the statistics of "Optimal Covering" at 3 levels: "STP", "Source (with target)", and "Target (with source)" for the indicated tissues. The quantity is the number of features which passed the 2% filter at the indicated level (e.g. STP, source and target.). After setting the numbers of solutions limit up to $10,000$ in the optimization program, the number of optimal coverings of each type for each tissue is reported. For instance, for breast cancer at the "STP" level, there are 12890 candidate STPs after 2 step filters, each optimal solution contains 73 STPs, 563 STPs involved in all solutions, and about 97.7% of breast cancer samples can be covered by every optimal covering. Finally, there are 16 core STPs that exist in every covering. Similar statistics are reported for all other tissue types considered in this study.

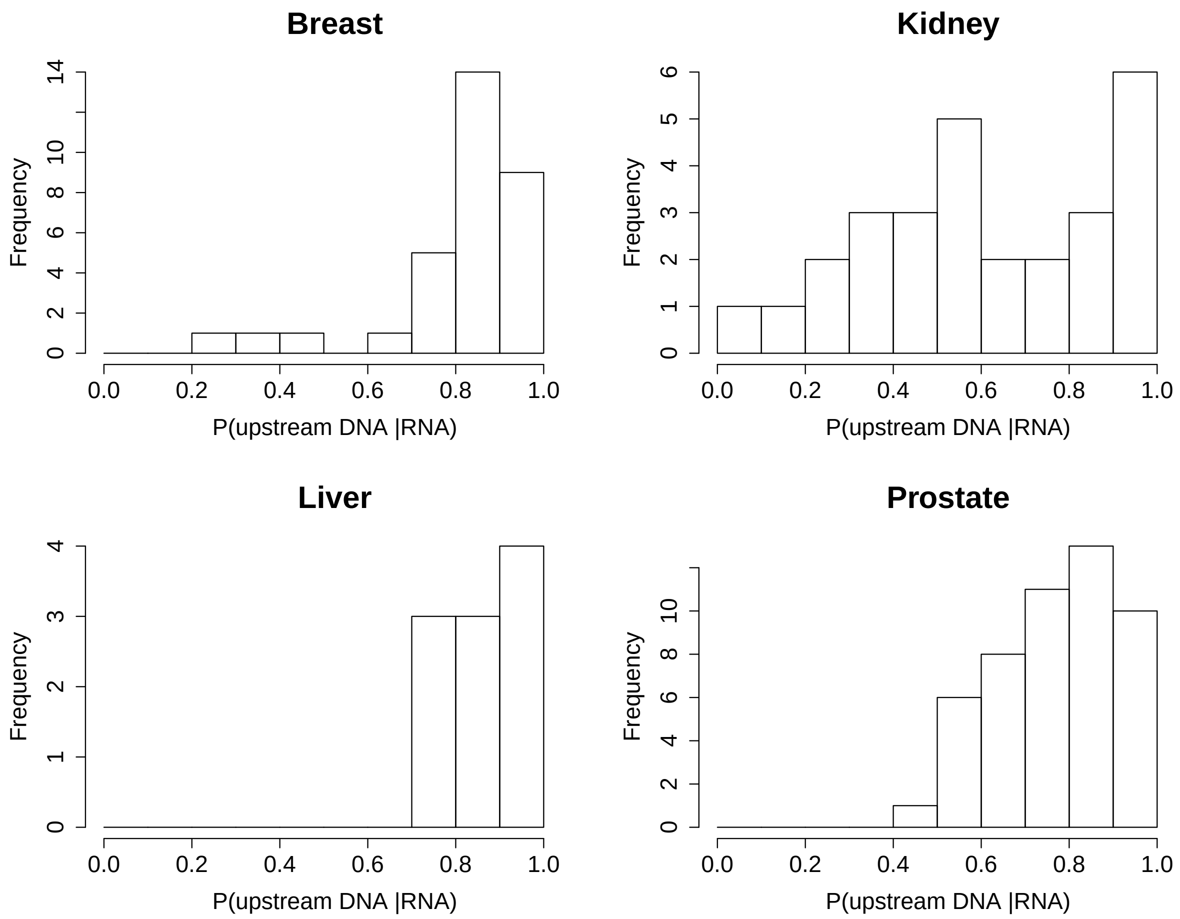

Figure A: P(**upstream DNA**|**RNA**) **for target core across tissues** This figure shows P(upstream DNA|RNA) for "core" at target level of 4 tissues (Breast, Kidney, Liver, and Prostate), where P(upstream DNA|RNA) is the fraction of samples with indicated target gene RNA-aberrant for which there exists some DNA-aberrant gene among its source. For all four tissues in the figure, P(upstream DNA|RNA) is usually large as between $0.7 - 1$.

1.2 Filters: 1% v.s 2% v.s 5%

Having settled on the choice of $k = 3$, we now proceed to consider the filter which omits rare events.

Recall from Section 3.2, we applied a 2% filter to remove rare events at each of three levels (STP, source, target); that is, the remaining STPs (respectively for sources, targets) after this procedure are aberrant in at least 2% of samples for a given tissue. Generally speaking, the selection of 2% is due to limitations of sample size. We usually have hundreds of samples per tissue, thus each STP (source, target) remaining after such a 2% cutoff is aberrant for at least 2 samples and usually more.

Here we conduct a further analysis and compare results between different percentage values (e.g., $1\%, 2\%, 5\%$) for this filter. Supplementary Figure B (below) demonstrates our findings, indicating that a 2% cutoff is a moderate choice based on the resulting covering statistics. For instance, with a 2% filter at source level, the size of optimal coverings is usually between 10-60, and the fraction of samples covered is above 90% for most tissues (Table F). Further, the core set contains both

well-known cancer genes and potential cancer related genes (see Tables L -P).

A thresholding at 1% on the other hand, even though the fraction of samples covered is higher than 2%, the core set contains many STPs (sources, targets) which are relatively more cumbersome to analyze since these STPs (sources, targets) are only aberrant for a small fraction of samples. For instance, in breast cancer with a 1% filter, about 29% of core STPs are aberrant for less than 2% of the samples and 35% of core sources are aberrant with some target for less than 2% of the samples. Further, if we use a 5% filter, while we can usually find those STPs (sources, target) which are well-known to be of interest in cancer biology and pathology, the fraction of samples covered decreases considerably. For instance in prostate cancer, with a 5% filter only 56.8% (64%, 63%) of samples are covered by the optimal covering at STP (source, target) level correspondingly. Together these statistics suggest a cutoff of 2% as the most reasonable choice.

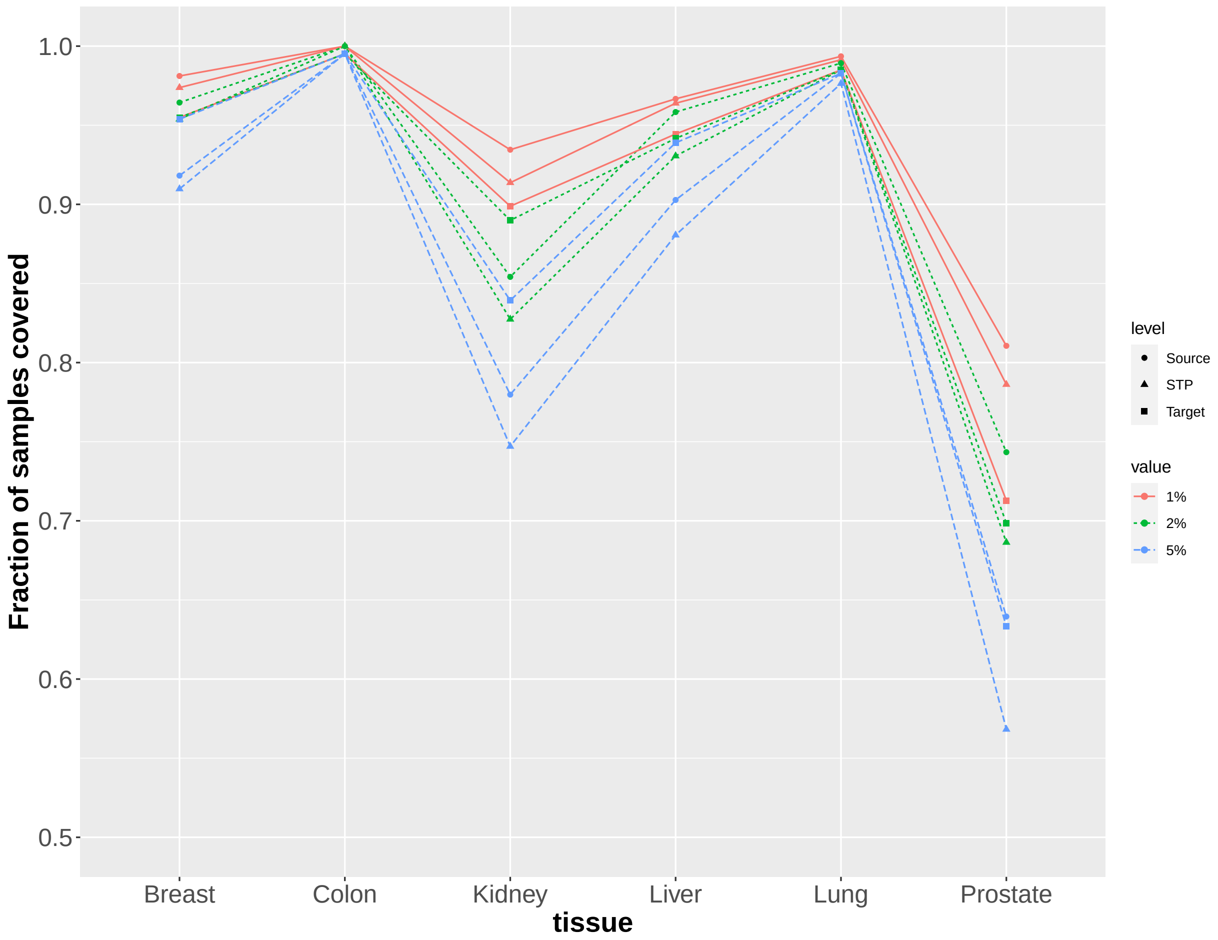

Figure B: Fraction of samples covered for different filter values. This figure shows the fraction of samples covered for different filter values at indicated tissues and levels (STP, Source, Target). There are 3 candidates values: $1\%, 2\%, 5\%$. For fixed tissue and level, as we increase the filter value, the fraction of samples covered decreases.

1.3 $L = 4$ v.s $L = 5$

Recall from Section 2.4, we described a greedy aggregation procedure to estimate an upper bound of the entropy of $\bar{Z} = (Z_j, j \in J)$, there is one parameter L which is the maximal size of subsets

that partition J. And we use $L = 4$ in our experiments. Our criterion here is that we require at least 2^L samples for reliable estimations. With L variables, there are 2^L parameters to estimate in computing entropy; requiring 2^L samples is then a minimum. It's also appropriate to use $L = 5$ if the sample size is reasonable. For instance, in breast cancer, the estimated entropy statistics for source level (source aberration with target) with $L = 4$ and $L = 5$ are very similar as shown in Table B. We see that the entropy estimated with $L = 4$ is within the range of confidence interval estimated with $L = 5$, and vise versa. However, for some other cancer subtypes like Tumor Status T2 of colon cancer, we only have 28 samples for which it is not reliable to estimate entropy with $L = 5$. For consistency and accuracy, we use $L = 4$ for all cancer subtypes.

Breast

Subtype	Value	N	L=4		L=5	
			Entropy	Conf.Interval	Entropy	Conf.Interval
All		953	17.02	[16.63, 17.40]	16.84	[16.43, 17.23]
PAM50	Basal-like	87	22.59	[21.34, 23.69]	22.07	[20.89, 23.13]
PAM50	HER2-enriched	55	18.95	[17.52, 20.14]	18.42	[17.10, 19.48]
PAM50	Luminal A	219	12.72	[11.96, 13.38]	12.44	[11.67, 13.13]
PAM50	Luminal B	121	17.43	[16.45, 18.34]	17.11	[16.15, 18.05]
ER Status	Negative	167	22.39	[21.36, 23.25]	22.06	[21.18, 22.95]
ER Status	Positive	569	15.51	[15.04, 15.97]	15.27	[14.81, 15.75]
Tumor Status	T1	249	13.56	[12.88, 14.21]	13.25	[12.59, 13.89]
Tumor Status	T2	551	18.25	[17.72, 18.74]	18.04	[17.54, 18.52]
Tumor Status	T3-T4	152	16.42	[15.53, 17.26]	16.13	[15.24, 16.95]
Lymph Node Status	Negative	446	17.35	[16.76, 17.89]	17.15	[16.57, 17.67]
Lymph Node Status	Positive	318	16.11	[15.45, 16.75]	15.89	[15.22, 16.52]

Table B: Entropy for source aberration with target with $L = 4$ and $L = 5$. This table provides detailed entropy statistics estimated with $L = 4$ and $L = 5$ for Breast cancer at source level. The estimated entropy statistics with $L = 4$ and $L = 5$ are very similar.

1.4 STP formation with Reactome

Here we present some further information regarding our use of Reactome for building STPs.

Reactome is a curated, open-source relational database of biological pathways and processes, encompassing proteins, metabolites, and their relationships. Overall, the Reactome network accounts for 336,214 interactions of 12 different types, occurring between 12,085 nodes (genes, proteins, complexes, and metabolites), which participate in 6,352 biological pathways. For our work, we restricted the analysis to gene regulatory motifs, excluding all metabolites, their modification, and their interactions. This resulted in a network encompassing a total of 110,686 interactions of 2 types ("controls state change of" and "controls expression of"), occurring between 6,725 genes participating in 1,400 biological pathways and processes. Source-target pairs were built from this network as explained in Section 2.2.2

1.5 Aberrations belonging to the cores

Our observations in the core sets re-iterate some know and expected facets of cancer biology. For example, *PTEN* (inactivation or deletion) [1] appears in the core signatures for breast, kidney, and prostate cancers, *PIK3CA* (mostly by mutation) [2] was found in breast and colon cancer, while *BRCA1* [3] was inactivated in breast and liver cancer. In addition, a number of aberrations affecting important cancer genes proved to be restricted to specific tissue of origin according to cell lineage and identity, and to canonical pathways of pathogenesis. For instance, *GATA3*, a transcription factor required for normal mammary gland development, and known to be altered in breast tumors [4], was only found in the core signature for breast cancer. Similarly, *APC*, the tumor suppressor gene mutated in familial colon cancer and most sporadic cases of the disease [5], was present only in the colon cancer core set. Finally, among other known cancer drivers, *VHL*, for instance, was part of the core set only in kidney cancer [6], *EGFR* and *BRAF* were recovered in lung cancer [7], and *CDKNB1* [8] and *FOXA1* [9, 10] in prostate cancer, according to expectations in line with known cancer biology and recent extensive analyses [11, 12].

Among the core target set, *FABP4* is a fatty acid binding protein playing an important role in glucose and lipid homeostasis [13] and has aberrant expression (with an upstream DNA alteration) in all cancer types except colon and lung, suggesting that the expression of this gene could serve as cancer biomarker. Similarly, and unexpectedly, several well-known cancer drivers, including *BRCA1, MSH2, TOP2A* and *EZH2*, were recovered among the core target genes. For instance, *EZH2* is a core target in prostate cancer [14] and *S100B* is one in breast tumors [15].

List of Tables

A Statistics of optimal coverings . . . 2
B Entropy for source aberration with target for Breast cancer . . . 5
C Basic statistics of interactions within k steps. . . . 9
D Basic statistics of interactions after χ^2 test. . . . 9
E Examples of STPs . . . 10
F Statistics of optimal coverings . . . 11
G Breast core STPs . . . 12
H Kidney core STPs . . . 12
I Liver core STPs . . . 13
J Lung core STPs . . . 13
K Prostate core STPs . . . 14
L Breast core source genes . . . 15
M Kidney core source genes . . . 16
N Liver core source genes . . . 17
O Lung core source genes . . . 18
P Prostate core source genes . . . 19
Q Breast core target genes . . . 20
R Kidney core target gene . . . 21
S Liver core target genes . . . 22
T Lung core target genes . . . 23
U Prostate core target genes . . . 25
V Aberration probabilities of selected targets with sources in PAM50 sub-types . . . 26
W Aberration probabilities of selected targets with sources for colon tumor groups . . . 26
X Aberration probabilities of selected targets with sources in liver tumor groups . . . 27
Y Aberration probabilities of selected targets with sources in lung tumor groups . . . 27
Z Aberration probabilities of selected targets with sources in prostate Gleason groups . 28
I Aberration probabilities of selected targets with sources in prostate primary Gleason groups . . . 28
II Entropy for source aberration with targetacross distinct tissues . . . 30
III Entropy for target aberration with source across distinct tissues . . . 32

List of Figures

A P(upstream DNA|RNA) . . . 3
B Covering rate for different filter values . . . 4
C Pair covering network for colon cancer . . . 33
D Pair covering network for kidney cancer . . . 34
E Pair covering network for liver cancer . . . 35
F Pair covering network for lung cancer . . . 36
G Pair covering network for prostate cancer . . . 37
H Annotated networks for PAM50 breast cancer sub-types . . . 38

I Annotated networks for lung cancer based groups based on smoking history 39
J Annotated networks for primary Gleason grade in prostate cancer 40
K Comparison of source sub-type coverings and pair probabilities across breast PAM50 classes 41
L Core set across tissues at the source level 42
M Core set across tissues at the target level 43
N Coding tree for breast ER status using DNA aberration with target 44
O Coding tree for lung smoking history using DNA aberration with target 45
P Coding tree for lung smoking history using RNA divergence with source 46

1.6 Supplementary Tables

	Interactions	Source Genes	Target Genes	Distinct Genes
$k = 1$	3730	510	598	1016
$k \leq 2$	34955	1915	598	2320
$k \leq 3$	272237	3124	598	3479
$k \leq 4$	1322385	3616	598	3952

Table C: Basic statistics of interactions within k steps. This table shows the number of interactions, source genes, target genes, and distinct genes within k steps, as retrieved from the Reactome network. For instance, in our experiment, when we set $k \leq 3$, there are 272237 interactions in total, and there are 3124 source genes, 598 target genes, and 3479 distinct genes in total.

Tissue	Filters	Interactions	Source Genes	Target Genes	Distinct Genes
Breast	After χ^2 test	17261	2130	421	2396
Colon	After χ^2 test	6297	1646	354	1892
Kidney	After χ^2 test	4870	1206	359	1485
Liver	After χ^2 test	7232	1688	421	1989
Lung	After χ^2 test	10908	1912	453	2218
Prostate	After χ^2 test	9301	1732	372	1985

Table D: Basic statistics of interactions after χ^2 test. This table shows the number of interactions, source genes, target genes, and distinct genes after χ^2 Test. The interactions remained are those which have P-value (uncorrected for multiple comparisons) less or equal than 0.05 between the binary DNA expression of source gene and ternary RNA expression of target gene.

Tissue	Pair	P(DNA&RNA)	P(DNA mut &RNA up)	P(DNA mut &RNA down)	P(DNA cnv del &RNA up)	P(DNA cnv del &RNA down)	P(DNA cnv dup &RNA up)	P(DNA cnv dup &RNA down)
Breast	*PIK3CA* ⇒ *S100B*	0.316	0.000	0.295	0.000	0.001	0.000	0.036
Colon	*APC*⇒ *AXIN2*	0.585	0.580	0.000	0.010	0.000	0.000	0.000
Kidney	*VHL*⇒ *CA9*	0.482	0.429	0.000	0.107	0.000	0.000	0.000
Liver	*TP53*⇒ *MYBL2*	0.308	0.294	0.000	0.022	0.000	0.000	0.000
Lung	*TP53*⇒ *TOP2A*	0.529	0.527	0.000	0.013	0.000	0.000	0.000
Prostate	*PTEN*⇒*TWIST1*	0.161	0.024	0.000	0.147	0.000	0.000	0.000

Table E: Examples of STPs. For each of six tissues, one example of a common STP $\lambda = (g \Rightarrow g')$ is shown. P(DNA&RNA) is the fraction of samples for which the indicated source gene is DNA-aberrant and indicated target gene is RNA-aberrant. P(DNA mut&RNA up) is the fraction of samples for which the indicated source gene is mutated and indicated target gene is over-expressed. P(DNA mut&RNA down) is the fraction of samples for which the indicated source gene is mutated and indicated target gene is under-expressed. P(DNA cnv det&RNA up) is the fraction of samples for which the indicated source gene has copy number deleted and target gene over-expressed. P(DNA cnv det&RNA down) is the fraction of samples for which the indicated source gene has copy number deleted and target gene under-expressed. P(DNA cnv dup&RNA up) is the fraction of samples for which the indicated source gene has copy number duplicated or more and target gene over-expressed. P(DNA cnv dup&RNA down) is the fraction of samples for which the indicated source gene has copy number duplicated or more and target gene under-expressed. To be noted that the sum of last 6 columns does not necessarily equal to P(DNA&RNA), since there could be two different type of aberrations occurring at same source genes for a fixed sample.

Tissue	Samples	Covering Type	Quantity	No. of solutions (up to 100000)	Size of solution	Total features in solution	Fraction of samples covered	Size of core set	Fraction of samples covered with core set
		STP	4026	100000	67	281	0.954	21	0.709
Breast	953	Source	690	100000	60	127	0.964	34	0.908
		Target	256	83712	53	87	0.955	35	0.917
		STP	1195	2353	11	101	1.000	4	0.807
Colon	207	Source	525	170	10	75	1.000	5	0.937
		Target	226	368	15	65	0.995	6	0.807
		STP	347	100000	26	67	0.827	12	0.732
Kidney	336	Source	133	256	28	37	0.854	21	0.830
		Target	176	18180	60	88	0.890	45	0.812
		STP	1198	100000	32	303	0.931	11	0.533
Liver	360	Source	460	9840	34	77	0.958	20	0.822
		Target	287	702	41	75	0.942	26	0.858
		STP	3154	100000	27	110	0.985	10	0.712
Lung	465	Source	908	140	25	42	0.989	19	0.974
		Target	350	15	29	34	0.985	26	0.981
		STP	430	100000	53	110	0.686	32	0.578
Prostate	491	Source	211	2620	53	69	0.743	42	0.707
		Target	160	192	72	81	0.699	66	0.686

Table F: Statistics of optimal coverings. This table shows the statistics of "Optimal Covering" at 3 levels: "STP", "Source (with target)", and "Target (with source)" for the indicated tissues. The quantity is the number of features which passed 2% filter at the indicated level (e.g. STP, source and target.). After setting the numbers of solutions limit up to $100,000$ in the optimization program, the number of optimal coverings of each type for each tissue is reported. For instance, for breast cancer at the "STP" level, there are 4026 candidate STPs after 2 step filters, there are $100,000$ solutions found from the optimization model, each solution contains 67 STPs, 281 STPs involved in all $100,000$ solutions, and about 95.4% of breast cancer samples can be covered by every optimal covering. Finally, there are 21 core STPs that exist in every covering, and 70.9% of breast cancer samples can be covered by such core set. Similar statistics are reported for all other tissue types considered in this study.

Pair	$P(\text{DNA}\,\&\,\text{RNA})$	$P(\text{DNA})$	$P(\text{RNA})$	$P(\text{RNA}\mid\text{DNA})$	$P(\text{DNA}\mid\text{RNA})$
PIK3CA ⇒ *MMP13*	0.222	0.356	0.574	0.625	0.388
BAG4 ⇒ *LIFR*	0.133	0.148	0.831	0.901	0.160
GATA3 ⇒ *CDC25C*	0.116	0.137	0.745	0.847	0.156
CLTC ⇒ *S100B*	0.102	0.111	0.838	0.915	0.121
CDH1 ⇒ *LIFR*	0.100	0.132	0.831	0.754	0.120
GATA3 ⇒ *LGALS3*	0.081	0.137	0.434	0.588	0.186
CDH1 ⇒ *KCTD6*	0.069	0.132	0.359	0.524	0.193
PTEN ⇒ *S100B*	0.067	0.093	0.838	0.719	0.080
PTEN ⇒ *FOXP3*	0.063	0.093	0.534	0.674	0.118
GAB2 ⇒ *SOD2*	0.048	0.077	0.508	0.630	0.095
IFT140 ⇒ *PTCH1*	0.039	0.055	0.526	0.712	0.074
CNOT3 ⇒ *CDC25C*	0.035	0.037	0.745	0.943	0.046
DNM3 ⇒ *MMP3*	0.031	0.108	0.207	0.291	0.152
PLXNA4 ⇒ *CDC25C*	0.028	0.029	0.745	0.964	0.038
MYB ⇒ *CDC25A*	0.028	0.039	0.509	0.730	0.056
AARS ⇒ *ACACB*	0.027	0.029	0.704	0.929	0.039
LAMA1 ⇒ *MMP1*	0.026	0.033	0.540	0.806	0.049
MMP9 ⇒ *MMP1*	0.026	0.036	0.540	0.735	0.049
PRKDC ⇒ *OPRD1*	0.025	0.088	0.193	0.286	0.130
MBTPS1 ⇒ *LPL*	0.025	0.030	0.614	0.828	0.041
MUC20 ⇒ *SAA1*	0.023	0.057	0.566	0.407	0.041

Table G: Breast core STPs. There are twenty-one "core" STPs which appear in every minimal covering of the breast samples. $P(\text{DNA}\,\&\,\text{RNA})$ is the fraction of samples for which the source gene g is DNA-aberrant and target gene g' is RNA-aberrant; $P(\text{DNA})$ is the fraction of samples satisfying source gene g is DNA-aberrant; $P(\text{RNA})$ is the fraction of samples with g' RNA-aberrant; $P(\text{RNA}\mid\text{DNA})$ is the fraction of DNA-aberrant samples for which g' is RNA-aberrant.

Pair	$P(\text{DNA}\,\&\,\text{RNA})$	$P(\text{DNA})$	$P(\text{RNA})$	$P(\text{RNA}\mid\text{DNA})$	$P(\text{DNA}\mid\text{RNA})$
VHL ⇒ *CA9*	0.482	0.485	0.967	0.994	0.498
PBRM1 ⇒ *BCL2*	0.223	0.429	0.446	0.521	0.500
PBRM1 ⇒ *ATM*	0.134	0.429	0.250	0.312	0.536
SLC12A2 ⇒ *EBI3*	0.113	0.164	0.557	0.691	0.203
BAP1 ⇒ *TP53AIP1*	0.083	0.202	0.289	0.412	0.289
RAF1 ⇒ *HEY1*	0.068	0.113	0.438	0.605	0.156
PBRM1 ⇒ *RAG1*	0.057	0.429	0.220	0.132	0.257
TTC37 ⇒ *CASP1*	0.045	0.051	0.536	0.882	0.083
ATM ⇒ *TNFRSF10D*	0.039	0.080	0.271	0.481	0.143
MTOR ⇒ *ITCH*	0.030	0.071	0.146	0.417	0.204
BAP1 ⇒ *CASP6*	0.027	0.202	0.062	0.132	0.429
RYR2 ⇒ *GZMB*	0.027	0.042	0.360	0.643	0.074

Table H: Kidney core STPs. There are twelve "core" STPs which appear in every minimal covering of the kidney samples. $P(\text{DNA}\,\&\,\text{RNA})$ is the fraction of samples for which the source gene g is DNA-aberrant and target gene g' is RNA-aberrant; $P(\text{DNA})$ is the fraction of samples satisfying source gene g is DNA-aberrant; $P(\text{RNA})$ is the fraction of samples with g' RNA-aberrant; $P(\text{RNA}\mid\text{DNA})$ is the fraction of DNA-aberrant samples for which g' is RNA-aberrant.

Pair	P(DNA & RNA)	P(DNA)	P(RNA)	P(RNA|DNA)	P(DNA|RNA)
CTNNB1 ⇒ *CXCL12*	0.261	0.269	0.897	0.969	0.291
CTNNB1 ⇒ *LCN2*	0.136	0.269	0.383	0.505	0.355
RB1 ⇒ *CHEK1*	0.100	0.111	0.506	0.900	0.198
BAP1 ⇒ *STEAP3*	0.064	0.069	0.725	0.920	0.088
HERC2 ⇒ *IGFBP3*	0.053	0.053	0.833	1.000	0.063
ALMS1 ⇒ *MSH2*	0.042	0.058	0.567	0.714	0.074
ANK1 ⇒ *HEY2*	0.036	0.078	0.322	0.464	0.112
BRCA1 ⇒ *TNFRSF10B*	0.033	0.047	0.367	0.706	0.091
CDC27 ⇒ *FGF2*	0.028	0.058	0.253	0.476	0.110
NCOR1 ⇒ *F7*	0.028	0.053	0.411	0.526	0.068
CLTC ⇒ *S1PR1*	0.025	0.069	0.256	0.360	0.098

Table I: Liver core STPs. There are eleven "core" STPs which appear in every minimal covering of the liver samples. $P(\text{DNA}\,\&\,\text{RNA})$ is the fraction of samples for which the source gene g is DNA-aberrant and target gene g' is RNA-aberrant; $P(\text{DNA})$ is the fraction of samples satisfying source gene g is DNA-aberrant; $P(\text{RNA})$ is the fraction of samples with g' RNA-aberrant; $P(\text{RNA}|\text{DNA})$ is the fraction of DNA-aberrant samples for which g' is RNA-aberrant.

Pair	P(DNA & RNA)	P(DNA)	P(RNA)	P(RNA|DNA)	P(DNA|RNA)
KRAS ⇒ *CD36*	0.335	0.351	0.914	0.957	0.367
ANK2 ⇒ *DLGAP5*	0.189	0.196	0.826	0.967	0.229
EGFR ⇒ *LPL*	0.159	0.168	0.832	0.949	0.191
SMARCA4 ⇒ *CHEK1*	0.092	0.095	0.766	0.977	0.121
KIF4B ⇒ *RUNX2*	0.058	0.069	0.613	0.844	0.095
LRRK2 ⇒ *TP53*	0.052	0.069	0.503	0.750	0.103
ALMS1 ⇒ *PTK6*	0.047	0.071	0.744	0.667	0.064
FRS2 ⇒ *IL23A*	0.047	0.065	0.527	0.733	0.090
BRAF ⇒ *CDKN1A*	0.026	0.105	0.108	0.245	0.240
FYN ⇒ *VIM*	0.024	0.028	0.798	0.846	0.030

Table J: Lung core STPs. There are ten "core" STPs which appear in every minimal covering of the lung samples. $P(\text{DNA}\,\&\,\text{RNA})$ is the fraction of samples for which the source gene g is DNA-aberrant and target gene g' is RNA-aberrant; $P(\text{DNA})$ is the fraction of samples satisfying source gene g is DNA-aberrant; $P(\text{RNA})$ is the fraction of samples with g' RNA-aberrant; $P(\text{RNA}|\text{DNA})$ is the fraction of DNA-aberrant samples for which g' is RNA-aberrant.

Pair	P(DNA & RNA)	P(DNA)	P(RNA)	P(RNA|DNA)	P(DNA|RNA)
PTEN ⇒ *TWIST1*	0.161	0.216	0.654	0.745	0.246
PTEN ⇒ *LGALS3*	0.151	0.216	0.550	0.698	0.274
FGF17 ⇒ *BNIP3L*	0.120	0.169	0.462	0.711	0.260
HDAC2 ⇒ *HEY2*	0.073	0.145	0.257	0.507	0.286
PTEN ⇒ *ITGBL1*	0.073	0.216	0.189	0.340	0.387
NRG1 ⇒ *PBX1*	0.071	0.116	0.409	0.614	0.174
FYN ⇒ *BOLA2*	0.067	0.134	0.365	0.500	0.184
DVL2 ⇒ *TWIST1*	0.057	0.067	0.654	0.848	0.087
FOXA1 ⇒ *MYL9*	0.047	0.081	0.381	0.575	0.123
SPTA1 ⇒ *MYL9*	0.045	0.063	0.381	0.710	0.118
DSCAM ⇒ *BNIP3L*	0.043	0.130	0.462	0.328	0.093
ZFHX3 ⇒ *DHFR*	0.039	0.126	0.163	0.306	0.237
STAT3 ⇒ *TWIST1*	0.037	0.039	0.654	0.947	0.056
PIK3CA ⇒ *MYL9*	0.035	0.061	0.381	0.567	0.091
SNAP91 ⇒ *PTGS2*	0.033	0.126	0.159	0.258	0.205
TP53 ⇒ *TP53*	0.031	0.179	0.055	0.170	0.556
DIS3 ⇒ *TNFRSF10C*	0.029	0.110	0.106	0.259	0.269
NRG1 ⇒ *CPT1A*	0.029	0.116	0.100	0.246	0.286
PTEN ⇒ *MMP9*	0.024	0.216	0.059	0.113	0.414
RAD17 ⇒ *TP53I3*	0.024	0.063	0.141	0.387	0.174
RFC3 ⇒ *BCL2*	0.024	0.071	0.175	0.343	0.140
MYC ⇒ *DHFR*	0.024	0.086	0.163	0.286	0.150
IL6ST ⇒ *ANXA2*	0.022	0.077	0.464	0.289	0.048
JAK1 ⇒ *BOLA2*	0.022	0.037	0.365	0.611	0.061
PIK3CA ⇒ *ITGBL1*	0.022	0.061	0.189	0.367	0.118
NUP205 ⇒ *PBX1*	0.022	0.022	0.409	1.000	0.055
ZFPM1 ⇒ *MAPKAPK5*	0.022	0.094	0.104	0.239	0.216
CAMK2G ⇒ *LGALS3*	0.020	0.022	0.550	0.909	0.037
CAST ⇒ *SCO2*	0.020	0.051	0.189	0.400	0.108
IRS2 ⇒ *LGALS3*	0.020	0.022	0.550	0.909	0.037
FANCD2 ⇒ *HIGD1A*	0.020	0.035	0.210	0.588	0.097
MYC ⇒ *BTG2*	0.020	0.086	0.116	0.238	0.175

Table K: Prostate core STPs. There are thirty-two "core" STPs which appear in every minimal covering of the prostate samples. $P(\mathrm{DNA}\,\&\,\mathrm{RNA})$ is the fraction of samples for which the source gene g is DNA-aberrant and target gene g' is RNA-aberrant; $P(\mathrm{DNA})$ is the fraction of samples satisfying source gene g is DNA-aberrant; $P(\mathrm{RNA})$ is the fraction of samples with g' RNA-aberrant; $P(\mathrm{RNA}|\mathrm{DNA})$ is the fraction of DNA-aberrant samples for which g' is RNA-aberrant.

Source	P(DNA & downstream RNA)	P(DNA)	P(downstream RNA|DNA)
PIK3CA	0.353	0.356	0.991
TP53	0.313	0.313	1.000
PTK2	0.164	0.165	0.994
BAG4	0.145	0.148	0.979
GATA3	0.134	0.137	0.977
CDH1	0.129	0.132	0.976
NCSTN	0.114	0.123	0.932
CLTC	0.111	0.111	1.000
NCOA2	0.103	0.103	1.000
MED1	0.100	0.101	0.990
PTEN	0.093	0.093	1.000
MUC20	0.056	0.057	0.981
DNM3	0.046	0.108	0.427
IFT140	0.042	0.055	0.769
BRCA1	0.037	0.038	0.972
CNOT3	0.037	0.037	1.000
ATM	0.036	0.042	0.850
GRIN2B	0.036	0.036	1.000
MMP9	0.035	0.036	0.971
TFDP1	0.035	0.035	1.000
FURIN	0.031	0.035	0.909
DSCAM	0.030	0.030	1.000
DUSP16	0.029	0.031	0.933
PIK3R1	0.029	0.029	1.000
AARS	0.028	0.029	0.964
PHLPP2	0.028	0.028	1.000
POM121	0.028	0.029	0.964
HIST1H3B	0.027	0.027	1.000
PIK3C2G	0.025	0.030	0.828
MBTPS1	0.025	0.030	0.828
PPARG	0.024	0.025	0.958
ITSN2	0.023	0.023	1.000
SLC27A5	0.023	0.030	0.759
FAM20C	0.021	0.021	1.000

Table L: Breast core source genes. There are thirty-four "core" source genes which appear in every minimal source covering of the breast samples. P(DNA) is the fraction of samples for which the indicated source gene is DNA-aberrant; P(DNA & downstream RNA) is the fraction of samples for which the indicated source gene is DNA-aberrant and there exists an RNA-aberrant gene among its targets. P(downstream RNA|DNA) is the fraction of the samples with the indicated source gene DNA-aberrant for which there exists some RNA-aberrant gene among its targets.

Source	P(DNA & downstream RNA)	P(DNA)	P(downstream RNA|DNA)
VHL	0.482	0.485	0.994
PBRM1	0.307	0.429	0.715
MAML1	0.179	0.179	1.000
NRG2	0.173	0.173	1.000
SLC12A2	0.113	0.164	0.691
CDC25C	0.095	0.167	0.571
RAF1	0.095	0.113	0.842
BAP1	0.092	0.202	0.456
ATM	0.065	0.080	0.815
MTOR	0.060	0.071	0.833
PTEN	0.048	0.048	1.000
TTC37	0.045	0.051	0.882
CDC27	0.030	0.057	0.526
RYR2	0.027	0.042	0.643
TP53	0.027	0.027	1.000
FGFR1	0.024	0.024	1.000
ROCK1	0.024	0.024	1.000
GRIN2B	0.021	0.021	1.000
JAK2	0.021	0.021	1.000
TEX15	0.021	0.021	1.000
TAF1	0.021	0.021	1.000

Table M: Kidney core source genes. There are twenty-one "core" source genes which appear in every minimal source covering of the kidney samples. $P(\text{DNA})$ is the fraction of samples for which the indicated source gene is DNA-aberrant; $P(\text{DNA}\,\&\,\text{downstream RNA})$ is the fraction of samples for which the indicated source gene is DNA-aberrant and there exists an RNA-aberrant gene among its targets. $P(\text{downstream RNA}|\text{DNA})$ is the fraction of the samples with the indicated source gene DNA-aberrant for which there exists some RNA-aberrant gene among its targets.

Source	P(DNA & downstream RNA)	P(DNA)	P(downstream RNA|DNA)
TP53	0.319	0.319	1.000
CTNNB1	0.269	0.269	1.000
RB1	0.108	0.111	0.975
NRG1	0.081	0.081	1.000
CCND1	0.075	0.075	1.000
BAP1	0.069	0.069	1.000
EHMT2	0.058	0.058	1.000
HERC2	0.053	0.053	1.000
PIK3CA	0.053	0.053	1.000
BRCA1	0.047	0.047	1.000
CDC27	0.047	0.058	0.810
ALMS1	0.044	0.058	0.762
IL6ST	0.033	0.036	0.923
CLTC	0.028	0.069	0.400
FANCB	0.028	0.028	1.000
PAN2	0.028	0.028	1.000
ITGB3	0.025	0.031	0.818
CAMK2A	0.022	0.028	0.800
CEP290	0.022	0.028	0.800
MTM1	0.022	0.028	0.800

Table N: Liver core source genes. There are twenty "core" source genes which appear in every minimal source covering of the liver samples. P(DNA) is the fraction of samples for which the indicated source gene is DNA-aberrant; P(DNA & downstream RNA) is the fraction of samples for which the indicated source gene is DNA-aberrant and there exists an RNA-aberrant gene among its targets. P(downstream RNA|DNA) is the fraction of the samples with the indicated source gene DNA-aberrant for which there exists some RNA-aberrant gene among its targets.

Source	P(DNA & downstream RNA)	P(DNA)	P(downstream RNA|DNA)
TP53	0.535	0.535	1.000
KRAS	0.351	0.351	1.000
SPTA1	0.303	0.303	1.000
ANK2	0.189	0.196	0.967
STK11	0.178	0.178	1.000
EGFR	0.168	0.168	1.000
RYR1	0.142	0.189	0.750
NUP155	0.127	0.127	1.000
PIK3C2B	0.105	0.110	0.961
SMARCA4	0.095	0.095	1.000
BRAF	0.092	0.105	0.878
MET	0.084	0.084	1.000
VWF	0.082	0.084	0.974
ALMS1	0.069	0.071	0.970
LRRK2	0.067	0.069	0.969
MTMR9	0.065	0.069	0.938
LAMB2	0.030	0.034	0.875
FYN	0.028	0.028	1.000
RAD50	0.024	0.026	0.917

Table O: Lung core source genes. There are nineteen "core" source genes which appear in every minimal source covering of the lung samples. $P(\text{DNA})$ is the fraction of samples for which the indicated source gene is DNA-aberrant; $P(\text{DNA \& downstream RNA})$ is the fraction of samples for which the indicated source gene is DNA-aberrant and there exists an RNA-aberrant gene among its targets. $P(\text{downstream RNA}|\text{DNA})$ is the fraction of the samples with the indicated source gene DNA-aberrant for which there exists some RNA-aberrant gene among its targets.

Source	P(DNA & downstream RNA)	P(DNA)	P(downstream RNA|DNA)
PTEN	0.210	0.216	0.972
FGF17	0.128	0.169	0.759
TP53	0.128	0.179	0.716
FYN	0.118	0.134	0.879
NRG1	0.112	0.116	0.965
SNAP91	0.102	0.126	0.806
ZFHX3	0.092	0.126	0.726
MYC	0.086	0.086	1.000
DIS3	0.069	0.110	0.630
FOXA1	0.067	0.081	0.825
IL6ST	0.065	0.077	0.842
NCOA2	0.059	0.063	0.935
ITGA2B	0.055	0.067	0.818
LYN	0.049	0.051	0.960
PDE7A	0.049	0.055	0.889
PIK3CA	0.049	0.061	0.800
RAD17	0.043	0.063	0.677
TSLP	0.041	0.049	0.833
POLK	0.039	0.051	0.760
ZFPM1	0.037	0.094	0.391
STAT3	0.037	0.039	0.947
JAK1	0.035	0.037	0.944
CDH1	0.033	0.059	0.552
KL	0.033	0.069	0.471
RFC3	0.033	0.071	0.457
CTNNB1	0.031	0.031	1.000
DYNC1H1	0.031	0.031	1.000
E2F4	0.031	0.035	0.882
ERBB4	0.029	0.033	0.875
CCNH	0.026	0.043	0.619
SGIP1	0.024	0.026	0.923
ACTN2	0.022	0.035	0.647
HSP90AA1	0.022	0.022	1.000
CAMK2G	0.020	0.022	0.909
CDK5RAP2	0.020	0.022	0.909
CDKN1B	0.020	0.069	0.294
GNG12	0.020	0.024	0.833
IL33	0.020	0.024	0.833
IL6R	0.020	0.020	1.000
IRS2	0.020	0.022	0.909
LRP5	0.020	0.022	0.909
NUP160	0.020	0.026	0.769

Table P: Prostate core source genes. There are forty-two "core" source genes which appear in every minimal source covering of the prostate samples. P(DNA) is the fraction of samples for which the indicated source gene is DNA-aberrant; P(DNA & downstream RNA) is the fraction of samples for which the indicated source gene is DNA-aberrant and there exists an RNA-aberrant gene among its targets. P(downstream RNA|DNA) is the fraction of the samples with the indicated source gene DNA-aberrant for which there exists some RNA-aberrant gene among its targets.

Target	P(RNA & upstream DNA)	P(RNA)	P(upstream DNA|RNA)
CDC25C	0.706	0.745	0.948
S100B	0.659	0.838	0.786
CDC25A	0.489	0.509	0.961
CXCL12	0.483	0.627	0.769
MMP1	0.467	0.540	0.864
PIK3R1	0.463	0.524	0.884
FOS	0.445	0.507	0.878
MMP13	0.441	0.574	0.768
FABP4	0.424	0.663	0.639
CD36	0.421	0.793	0.530
FOXO1	0.416	0.687	0.605
BRCA1	0.410	0.460	0.893
RHOU	0.410	0.663	0.619
BCL6	0.393	0.576	0.683
PLAGL1	0.376	0.535	0.702
LIFR	0.367	0.831	0.442
S1PR1	0.347	0.409	0.849
E2F7	0.347	0.362	0.959
SOCS2	0.341	0.437	0.781
MYL9	0.292	0.523	0.558
PTCH1	0.285	0.526	0.543
CASP1	0.282	0.366	0.771
CASP6	0.247	0.375	0.658
VIM	0.240	0.346	0.694
KCTD6	0.232	0.359	0.646
IFNB1	0.230	0.346	0.664
JAG1	0.220	0.319	0.691
MLH1	0.195	0.242	0.805
EPO	0.195	0.267	0.732
PTK6	0.172	0.370	0.465
RUNX2	0.159	0.255	0.626
RAG1	0.095	0.191	0.500
ITGAL	0.095	0.183	0.523
IFNA10	0.050	0.057	0.889
HIGD1A	0.045	0.084	0.537

Table Q: Breast core target genes. There are thirty-five "core" target genes which appear in every minimal target covering of the breast samples. P(RNA) is the fraction of samples for which the indicated target gene is RNA-aberrant; P(RNA & upstream DNA) is the fraction of samples for which the indicated target gene is RNA-aberrant and there exists an DNA-aberrant gene among its sources. P(upstream DNA|RNA) is the fraction of the samples with the indicated gene RNA-aberrant for which at least one of its sources is DNA-aberrant.

Target	P(RNA & upstream DNA)	P(RNA)	P(upstream DNA|RNA)
CA9	0.503	0.967	0.520
HIGD1A	0.473	0.929	0.510
BCL2	0.390	0.446	0.873
FABP7	0.223	0.881	0.253
EBI3	0.199	0.557	0.358
BAX	0.170	0.429	0.396
ATM	0.170	0.250	0.679
NDN	0.146	0.280	0.521
STEAP3	0.146	0.250	0.583
TP53AIP1	0.131	0.289	0.454
BBC3	0.122	0.277	0.441
HEY1	0.113	0.438	0.259
LIFR	0.092	0.402	0.230
TGFA	0.080	0.646	0.124
TNFRSF10D	0.080	0.271	0.297
AXIN1	0.080	0.268	0.300
BRCA1	0.080	0.280	0.287
FANCI	0.074	0.339	0.219
AIFM2	0.071	0.098	0.727
POU2F1	0.068	0.098	0.697
ZEB1	0.065	0.152	0.431
DDB2	0.065	0.964	0.068
MMP9	0.065	0.384	0.171
APAF1	0.060	0.089	0.667
FABP4	0.060	0.074	0.800
BRWD1	0.054	0.077	0.692
FOXO3	0.054	0.107	0.500
FGF2	0.051	0.095	0.531
NFE2	0.051	0.092	0.548
HEY2	0.051	0.485	0.104
GZMB	0.045	0.360	0.124
BCL2L1	0.042	0.068	0.609
NPAS2	0.039	0.065	0.591
TNFRSF10C	0.036	0.051	0.706
CDC25A	0.033	0.033	1.000
GADD45A	0.033	0.048	0.688
HGF	0.033	0.045	0.733
LGALS3	0.033	0.042	0.786
GCG	0.030	0.042	0.714
EPO	0.030	0.253	0.118
PIM1	0.027	0.027	1.000
EBAG9	0.027	0.062	0.429
SPP1	0.027	0.101	0.265
MMP1	0.024	0.140	0.170
HES1	0.021	0.065	0.318

Table R: Kidney core target genes. There are forty-five "core" target genes which appear in every minimal target covering of the kidney samples. $P(\text{RNA})$ is the fraction of samples for which the indicated target gene is RNA-aberrant; $P(\text{RNA}\,\&\,\text{upstream DNA})$ is the fraction of samples for which the indicated target gene is RNA-aberrant and there exists an DNA-aberrant gene among its sources. $P(\text{upstream DNA}|\text{RNA})$ is the fraction of the samples with the indicated gene RNA-aberrant for which at least one of its sources is DNA-aberrant.

Target	P(RNA & upstream DNA)	P(RNA)	P(upstream DNA|RNA)
MSH2	0.492	0.567	0.868
STEAP3	0.469	0.725	0.648
IGFBP3	0.436	0.833	0.523
FANCD2	0.422	0.481	0.879
CXCL12	0.367	0.897	0.409
MDC1	0.342	0.417	0.820
PPARGC1A	0.314	0.414	0.758
BNIP3L	0.261	0.406	0.644
LCN2	0.258	0.383	0.674
KLK2	0.250	0.361	0.692
CA9	0.244	0.511	0.478
TGFA	0.206	0.481	0.428
MYC	0.178	0.206	0.865
BGLAP	0.178	0.239	0.744
HEY2	0.164	0.322	0.509
TP53I3	0.164	0.281	0.584
KLK3	0.131	0.194	0.671
PCBP4	0.128	0.236	0.541
HEYL	0.111	0.231	0.482
CASP6	0.106	0.164	0.644
SMAD6	0.106	0.181	0.585
TRIAP1	0.103	0.111	0.925
FABP4	0.094	0.200	0.472
SOD1	0.078	0.347	0.224
NOS2	0.067	0.153	0.436
CLDN5	0.042	0.058	0.714

Table S: Liver core target genes. There are twenty-six "core" target genes which appear in every minimal target covering of the liver samples. P(RNA) is the fraction of samples for which the indicated target gene is RNA-aberrant; P(RNA & upstream DNA) is the fraction of samples for which the indicated target gene is RNA-aberrant and there exists an DNA-aberrant gene among its sources. P(upstream DNA|RNA) is the fraction of the samples with the indicated gene RNA-aberrant for which at least one of its sources is DNA-aberrant.

Target	P(RNA & upstream DNA)	P(RNA)	P(upstream DNA|RNA)
CDC25C	0.744	0.858	0.867
CHEK1	0.682	0.766	0.890
IHH	0.660	0.933	0.707
MYBL2	0.641	0.787	0.814
TOP2A	0.641	0.923	0.695
VIM	0.626	0.798	0.784
SALL4	0.563	0.701	0.804
PTK6	0.501	0.744	0.673
PPARGC1A	0.467	0.514	0.908
ITCH	0.402	0.484	0.831
TNFRSF18	0.372	0.518	0.718
FGF2	0.368	0.458	0.803
AIFM2	0.359	0.376	0.954
BCL2L14	0.346	0.378	0.915
E2F7	0.344	0.351	0.982
TFF1	0.329	0.424	0.777
CEBPA	0.301	0.383	0.787
SOCS2	0.301	0.353	0.854
PPARG	0.228	0.383	0.596
RORC	0.222	0.232	0.954
PBX1	0.211	0.385	0.547
BCL2L11	0.153	0.163	0.934
ITGA5	0.144	0.342	0.421
NOS2	0.047	0.058	0.815
SERPINB13	0.026	0.028	0.923
ABCB4	0.024	0.325	0.073

Table T: Lung core target genes. There are twenty-six "core" target genes which appear in every minimal target covering of the lung samples. $P(\text{RNA})$ is the fraction of samples for which the indicated target gene is RNA-aberrant; $P(\text{RNA}\,\&\,\text{upstream DNA})$ is the fraction of samples for which the indicated target gene is RNA-aberrant and there exists an DNA-aberrant gene among its sources. $P(\text{upstream DNA}|\text{RNA})$ is the fraction of the samples with the indicated gene RNA-aberrant for which at least one of its sources is DNA-aberrant.

Target	P(RNA & upstream DNA)	P(RNA)	P(upstream DNA|RNA)
LGALS3	0.316	0.550	0.574
MYL9	0.293	0.381	0.770
EZH2	0.271	0.369	0.735
BNIP3L	0.246	0.462	0.533
TWIST1	0.224	0.654	0.343
PBX1	0.189	0.409	0.463
HIGD1A	0.181	0.210	0.864
CCNA2	0.171	0.253	0.677
CDC25C	0.155	0.171	0.905
HEY2	0.149	0.257	0.579
ITGBL1	0.134	0.189	0.710
PTGS2	0.132	0.159	0.833
PMS2	0.128	0.196	0.656
BAX	0.124	0.171	0.726
TP53I3	0.122	0.141	0.870
TOP2A	0.120	0.177	0.678
CDC25A	0.118	0.191	0.617
TIMP1	0.118	0.320	0.369
DHFR	0.112	0.163	0.688
BOLA2	0.110	0.365	0.302
SCO2	0.110	0.189	0.581
APP	0.108	0.269	0.402
MSH2	0.106	0.116	0.912
BCL2	0.102	0.175	0.581
ARID3A	0.100	0.106	0.942
TNFRSF10C	0.096	0.106	0.904
FOS	0.094	0.124	0.754
FANCC	0.092	0.110	0.833
RAG1	0.086	0.141	0.609
ZIC3	0.086	0.189	0.452
CDKN1A	0.084	0.086	0.976
RRM2B	0.075	0.100	0.755
FABP4	0.073	0.122	0.600
MAPKAPK5	0.073	0.104	0.706
CCNE1	0.071	0.098	0.729
BRCA1	0.071	0.077	0.921
HIF1A	0.065	0.132	0.492
PLK1	0.065	0.206	0.317
BCL6	0.063	0.081	0.775
TNFRSF18	0.057	0.112	0.509
ATR	0.057	0.096	0.596
HSP90AA1	0.057	0.130	0.438
CPT1A	0.055	0.100	0.551
RBL1	0.047	0.084	0.561
PKLR	0.039	0.092	0.422
VIM	0.039	0.077	0.500
IFNB1	0.039	0.071	0.543
MDM2	0.037	0.061	0.600
NDN	0.037	0.043	0.857
PTPN9	0.037	0.090	0.409

IL2RA	0.035	0.049	0.708
EBI3	0.035	0.045	0.773
NANOG	0.035	0.043	0.810
HMGCS1	0.035	0.059	0.586
PTK6	0.033	0.051	0.640
PLAGL1	0.031	0.033	0.938
GLI1	0.031	0.061	0.500
ITGAL	0.026	0.033	0.813
NEUROG3	0.026	0.035	0.765
CSN2	0.024	0.043	0.571
MLH1	0.022	0.024	0.917
CCNB2	0.022	0.051	0.440
CCND1	0.020	0.022	0.909
SOCS4	0.020	0.020	1.000
IGFBP3	0.020	0.057	0.357
UGT1A9	0.020	0.077	0.263

Table U: Prostate core target genes. There are sixty-six "core" target genes which appear in every minimal target covering of the prostate samples. $P(\text{RNA})$ is the fraction of samples for which the indicated target gene is RNA-aberrant; $P(\text{RNA}\,\&\,\text{upstream DNA})$ is the fraction of samples for which the indicated target gene is RNA-aberrant and there exists an DNA-aberrant gene among its sources. $P(\text{upstream DNA}|\text{RNA})$ is the fraction of the samples with the indicated gene RNA-aberrant for which at least one of its sources is DNA-aberrant.

	Luminal A	Luminal B	HER2-enriched	Basal-like
CDC25A	0.228	0.694	0.800	0.943
CD36	0.228	0.512	0.800	0.770
S100B	0.685	0.760	0.982	0.402
MMP1	0.329	0.545	0.855	0.747
PLAGL1	0.370	0.669	0.582	0.172
CDC25C	0.516	0.901	0.964	0.977
E2F7	0.160	0.595	0.436	0.632
FABP4	0.260	0.570	0.745	0.667
EPO	0.132	0.289	0.564	0.115
FOS	0.352	0.645	0.818	0.713
BCL6	0.292	0.463	0.745	0.586
PTK6	0.142	0.223	0.527	0.126
BRCA1	0.269	0.686	0.418	0.540
FOXO1	0.269	0.570	0.673	0.494
IFNB1	0.119	0.223	0.309	0.517
MMP13	0.475	0.463	0.709	0.299
SOCS2	0.237	0.413	0.545	0.609
PIK3R1	0.311	0.603	0.527	0.690
CXCL12	0.406	0.570	0.764	0.701
S1PR1	0.242	0.504	0.545	0.540
LIFR	0.279	0.430	0.582	0.310
RHOU	0.406	0.529	0.527	0.253

Table V: Aberration probabilities of selected targets with sources in PAM50 sub-types. Aberration probabilities for PAM50 sub-types for targets with source selected from the union of the target coverings; targets with source were required to have at least probability 0.4 in at least one sub-type.

	CRIS-A	CRIS-B	CRIS-C	CRIS-D	CRIS-E
TNFRSF10B	0.739	0.783	0.200	0.500	0.645
AXIN2	0.391	0.435	0.714	0.893	0.742
MYBL2	0.022	0.087	0.457	0.357	0.419
PDX1	0.848	0.826	0.600	0.464	0.677
SALL4	0.739	0.826	0.457	0.643	0.645
PERP	0.717	0.826	0.571	0.643	0.806

Table W: Aberration probabilities of selected targets with sources for colon tumor groups. Targets with sources were selected by requiring a probability of 0.4 or more in at least one group.

	G1	G2	G3
STEAP3	0.240	0.402	0.630
FANCD2	0.220	0.385	0.555
MSH2	0.340	0.425	0.622
MDC1	0.260	0.293	0.445
IGFBP3	0.300	0.483	0.437
PPARGC1A	0.220	0.282	0.395
LCN2	0.240	0.236	0.303
CXCL12	0.380	0.356	0.387

Table X: Aberration probabilities of selected targets with sources in liver tumor groups. Targets with source were selected requiring a probability of 0.2 or more in at least one group.

	Smoker	Recently Reformed	Reformed	Non Smoker
AIFM2	0.465	0.371	0.367	0.074
CHEK1	0.814	0.774	0.483	0.556
BCL2L14	0.372	0.355	0.483	0.630
PPARGC1A	0.558	0.323	0.350	0.481
PTK6	0.535	0.274	0.417	0.444
E2F7	0.465	0.306	0.217	0.370
TFF1	0.395	0.403	0.317	0.185
CDC25C	0.814	0.774	0.667	0.593
MYBL2	0.698	0.677	0.600	0.481
FGF2	0.488	0.323	0.317	0.296
TOP2A	0.674	0.613	0.617	0.481
VIM	0.721	0.629	0.683	0.556
IHH	0.767	0.613	0.633	0.630
ITCH	0.488	0.435	0.367	0.333
TNFRSF18	0.465	0.339	0.333	0.333
SALL4	0.535	0.500	0.550	0.444

Table Y: Aberration probabilities of selected targets with sources in lung tumor groups. Targets with source were selected requiring a probability of 0.4 or more in at least one group.

	3+3	3+4	4+3	4+4	>(4+4)
EZH2	0.045	0.172	0.202	0.347	0.460
MYL9	0.068	0.221	0.263	0.449	0.410
CCNA2	0.045	0.041	0.111	0.184	0.367
CDC25C	0.000	0.055	0.141	0.143	0.324
BNIP3L	0.091	0.193	0.242	0.204	0.374
LGALS3	0.273	0.262	0.343	0.245	0.396
TWIST1	0.159	0.193	0.232	0.163	0.302

Table Z: Aberration probabilities of selected targets with sources in prostate Gleason groups. Targets with source were selected requiring a probability of 0.2 or more in at least one group.

	3	4	5
CCNA2	0.051	0.200	0.490
CDC25C	0.046	0.180	0.469
TOP2A	0.026	0.131	0.429
EZH2	0.148	0.310	0.551
BNIP3L	0.168	0.253	0.510
MYL9	0.189	0.343	0.449
CDC25A	0.092	0.098	0.306
PBX1	0.153	0.196	0.306
LGALS3	0.265	0.335	0.408

Table I: Aberration probabilities of selected targets with sources in prostate primary Gleason groups. Targets with source were selected requiring a probability of 0.2 or more in at least one group.

Subtype	Value	N	Entropy	Conf. Interval
		**Breast**		
All		953	17.02	[16.63, 17.40]
PAM50	Basal-like	87	22.59	[21.34, 23.69]
PAM50	HER2-enriched	55	18.95	[17.52, 20.14]
PAM50	Luminal A	219	12.72	[11.96, 13.38]
PAM50	Luminal B	121	17.43	[16.45, 18.34]
ER Status	Negative	167	22.39	[21.36, 23.25]
ER Status	Positive	569	15.51	[15.04, 15.97]
Tumor Status	T1	249	13.56	[12.88, 14.21]
Tumor Status	T2	551	18.25	[17.72, 18.74]
Tumor Status	T3-T4	152	16.42	[15.53, 17.26]
Lymph Node Status	Negative	446	17.35	[16.76, 17.89]
Lymph Node Status	Positive	318	16.11	[15.45, 16.75]
		**Colon**		
All		207	6.20	[5.92, 6.46]
Stage	I-II	113	6.52	[6.13, 6.87]
Stage	III-IV	85	5.63	[5.22, 5.99]
Tumor Status	T2	28	5.72	[5.01, 6.37]
Tumor Status	T3-T4	173	6.22	[5.91, 6.51]
Lymph Node Status	Negative	122	6.48	[6.14, 6.83]
Lymph Node Status	Positive	85	5.72	[5.28, 6.13]
		**Kidney**		
All		336	8.25	[7.86, 8.62]
Stage	I	178	7.61	[7.10, 8.09]
Stage	II-IV	157	8.66	[8.08, 9.22]
Tumor Status	T1	184	7.65	[7.15, 8.14]
Tumor Status	T2-T4	152	8.60	[7.98, 9.18]
		**Liver**		
All		360	10.93	[10.45, 11.38]
Stage	I	168	10.35	[9.67, 10.90]
Stage	II	85	11.01	[10.10, 11.83]
Stage	III-IV	81	10.12	[9.26, 10.87]
Histology	G1	50	9.28	[8.10, 10.30]
Histology	G2	174	10.85	[10.23, 11.45]
Histology	G3-G4	131	10.68	[9.94, 11.33]

Subtype	Value	N	Entropy	Conf. Interval
Tumor Status	T1	178	10.33	[9.70, 10.90]
Tumor Status	T2	93	10.91	[10.06, 11.69]
Tumor Status	T3-T4	86	10.77	[9.87, 11.64]
		**Lung**		
All		465	11.65	[11.30, 11.97]
Stage	I	241	11.67	[11.21, 12.09]
Stage	II	110	11.57	[10.96, 12.18]
Stage	III-IV	91	10.67	[9.94, 11.36]
Smoking history	Ancien	87	9.71	[8.95, 10.31]
Smoking history	Recent	105	12.33	[11.64, 12.97]
Tumor Status	T1	151	10.51	[9.95, 10.98]
Tumor Status	T2	252	11.83	[11.40, 12.25]
Tumor Status	T3-T4	60	12.50	[11.70, 13.28]
Lymph Node Status	Negative	296	11.91	[11.46, 12.30]
Lymph Node Status	Positive	158	11.03	[10.51, 11.56]

Table II: Entropy for source aberration with target across distinct tissues. Entropy estimation (upper bound) on source aberration with target for several tissue types and tumor subtypes. N is the total number of samples available in the given subtype.

Subtype	Value	N	Entropy	Conf. Interval
		**Breast**		
All		953	39.03	[38.72, 39.35]
PAM50	Basal-like	87	33.22	[32.31, 33.97]
PAM50	HER2-enriched	55	35.05	[33.97, 35.87]
PAM50	Luminal A	219	34.22	[33.53, 34.88]
PAM50	Luminal B	121	38.46	[37.72, 39.13]
ER Status	Negative	167	37.11	[36.39, 37.78]
ER Status	Positive	569	38.41	[38.01, 38.76]
Tumor Status	T1	249	36.31	[35.69, 36.90]
Tumor Status	T2	551	39.88	[39.49, 40.27]
Tumor Status	T3-T4	152	37.77	[36.99, 38.50]
Lymph Node Status	Negative	446	38.96	[38.53, 39.34]
Lymph Node Status	Positive	318	38.18	[37.63, 38.69]
		**Colon**		
All		207	12.40	[12.11, 12.68]
Stage	I-II	113	12.07	[11.65, 12.44]
Stage	III-IV	85	12.28	[11.80, 12.77]
Tumor Status	T2	28	11.26	[10.47, 11.93]
Tumor Status	T3-T4	173	12.43	[12.11, 12.74]
Lymph Node Status	Negative	122	12.11	[11.70, 12.53]
Lymph Node Status	Positive	85	12.35	[11.91, 12.76]
		**Kidney**		
All		336	20.88	[20.19, 21.48]
Stage	I	178	18.68	[17.81, 19.44]
Stage	II-IV	157	22.47	[21.50, 23.32]
Tumor Status	T1	184	18.80	[17.90, 19.62]
Tumor Status	T2-T4	152	22.33	[21.36, 23.30]
		**Liver**		
All		360	26.05	[25.58, 26.50]
Stage	I	168	24.97	[24.21, 25.58]
Stage	II	85	25.59	[24.62, 26.36]
Stage	III-IV	81	25.65	[24.65, 26.47]
Histology	G1	50	20.82	[19.61, 21.86]
Histology	G2	174	25.27	[24.60, 25.86]
Histology	G3-G4	131	26.76	[26.01, 27.43]

Subtype	Value	N	Entropy	Conf. Interval
Tumor Status	T1	178	24.92	[24.26, 25.56]
Tumor Status	T2	93	25.70	[24.74, 26.48]
Tumor Status	T3-T4	86	26.16	[25.22, 26.97]
**Lung**				
All		465	21.12	[20.83, 21.42]
Stage	I	241	21.17	[20.73, 21.53]
Stage	II	110	20.43	[19.81, 20.94]
Stage	III-IV	91	20.69	[20.00, 21.31]
Smoking history	Ancien	87	19.92	[19.22, 20.53]
Smoking history	Recent	105	20.94	[20.32, 21.47]
Tumor Status	T1	151	20.39	[19.86, 20.91]
Tumor Status	T2	252	21.25	[20.83, 21.60]
Tumor Status	T3-T4	60	20.47	[19.74, 21.07]
Lymph Node Status	Negative	296	21.20	[20.82, 21.55]
Lymph Node Status	Positive	158	20.52	[20.01, 21.02]
**Prostate**				
All		491	25.88	[25.23, 26.47]
Gleason	6	45	14.44	[12.77, 15.68]
Gleason	7	244	20.80	[19.90, 21.61]
Gleason	8	63	25.29	[23.61, 26.69]
Gleason	9	135	31.57	[30.41, 32.53]
Primary	3	196	17.53	[16.63, 18.37]
Primary	4	245	28.12	[27.21, 29.02]
Primary	5	49	32.13	[30.25, 33.54]
Tumor Status	T2	186	19.72	[18.86, 20.63]
Tumor Status	T3-T4	298	28.38	[27.57, 29.12]
Lymph Node Status	Negative	342	24.57	[23.78, 25.27]
Lymph Node Status	Positive	77	30.87	[29.43, 32.18]

Table III: Entropy for target aberration with source across distinct tissues. Entropy estimation (upper bound) on target aberration with source for several tissue types and subtypes.

1.7 Supplementary Figures

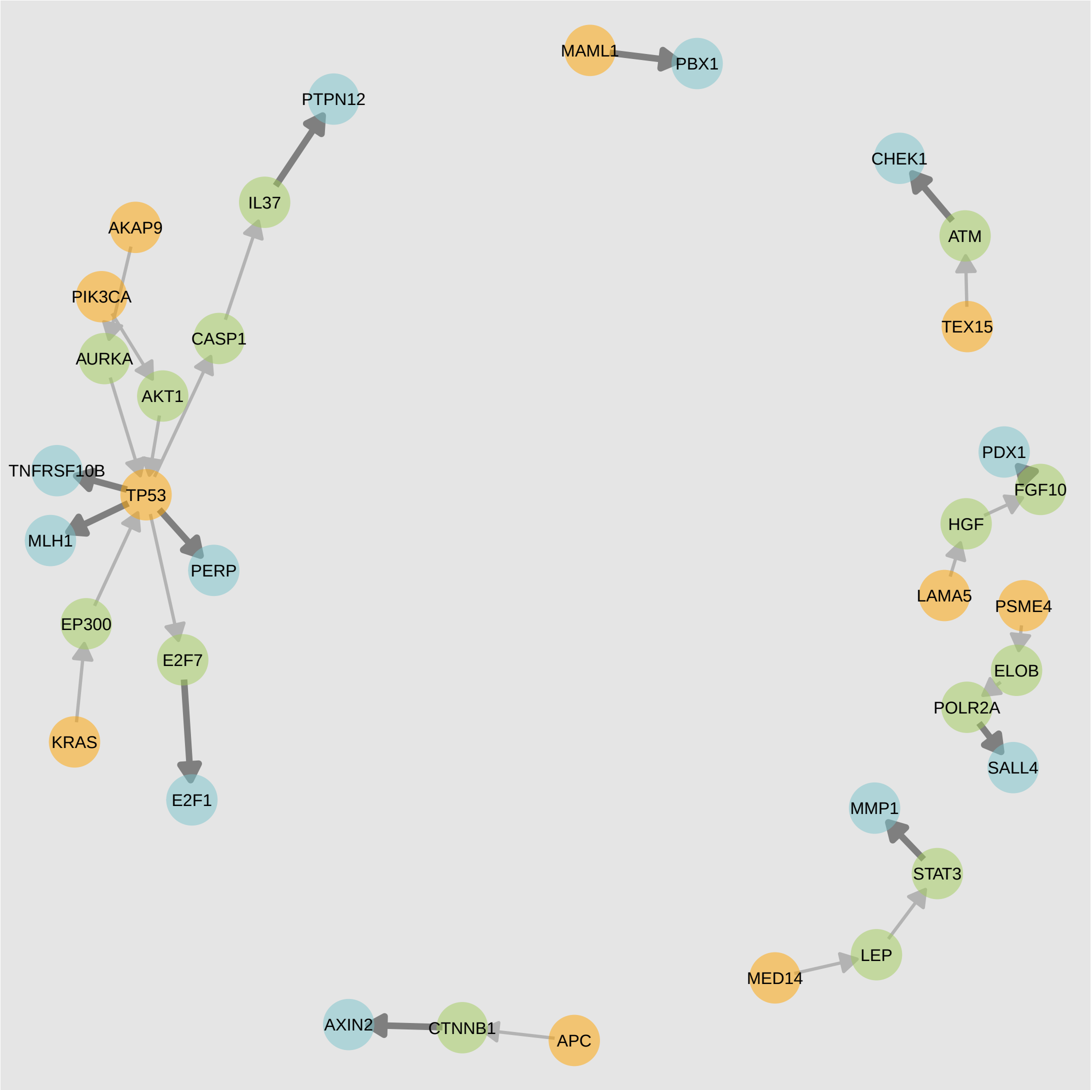

Figure C: Pair covering network for colon cancer. The graphical network here shows a selected pair covering obtained for colon tumors; sources are shown in orange, targets in blue, and the intermediary genes in green. Strong connections are shown in bold edges. Many genes known to play an important role in colon tumorigenesis such as *TP53* and *KRAS* can be seen in the network.

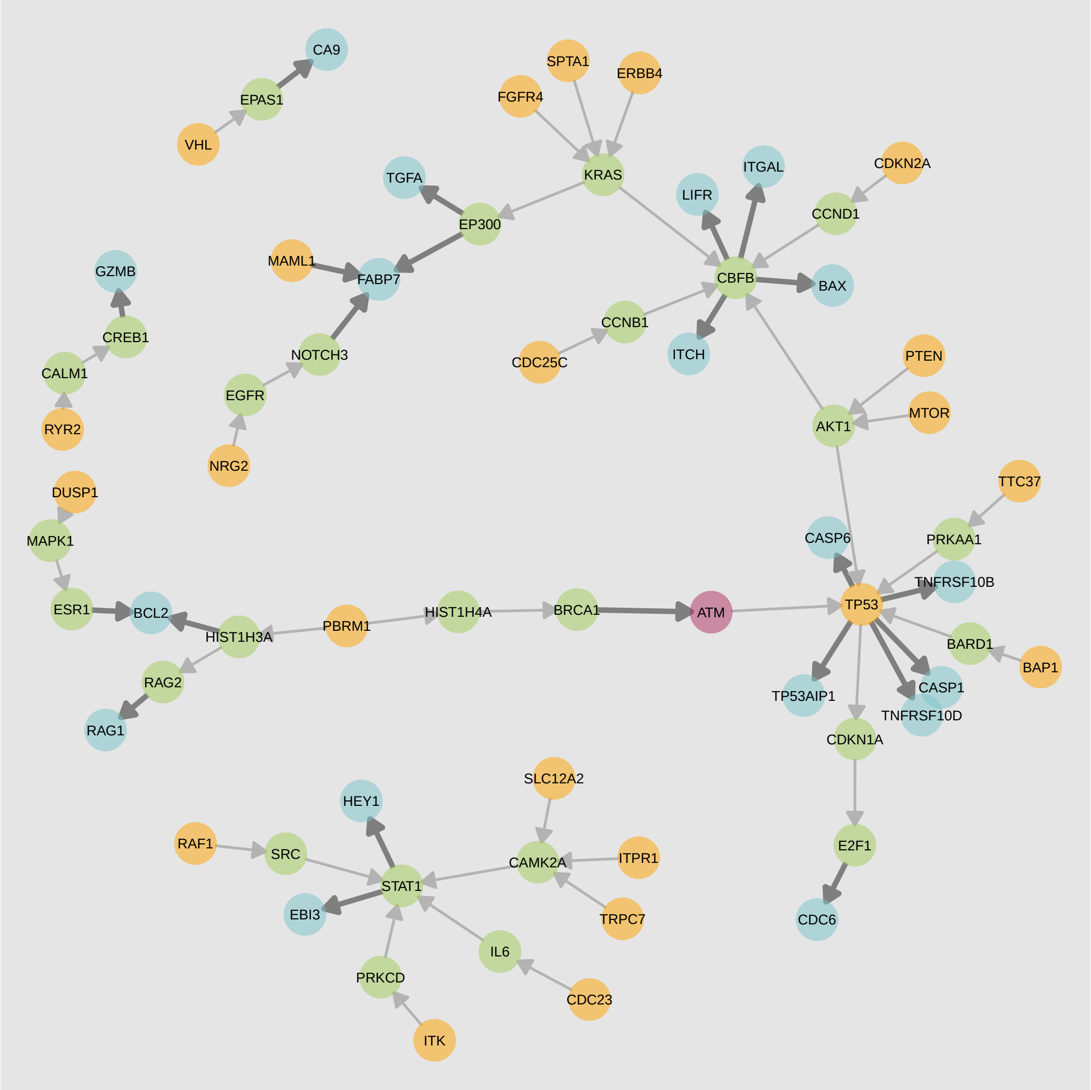

Figure D: Pair covering network for kidney cancer. A pair covering obtained for kidney renal clear cell carcinoma (KIRC) is shown as a network with sources are shown in orange, targets in blue, and the intermediary genes in green. Genes that are both source and a targets, are shown in red. Strong connections are shown in bold edges.

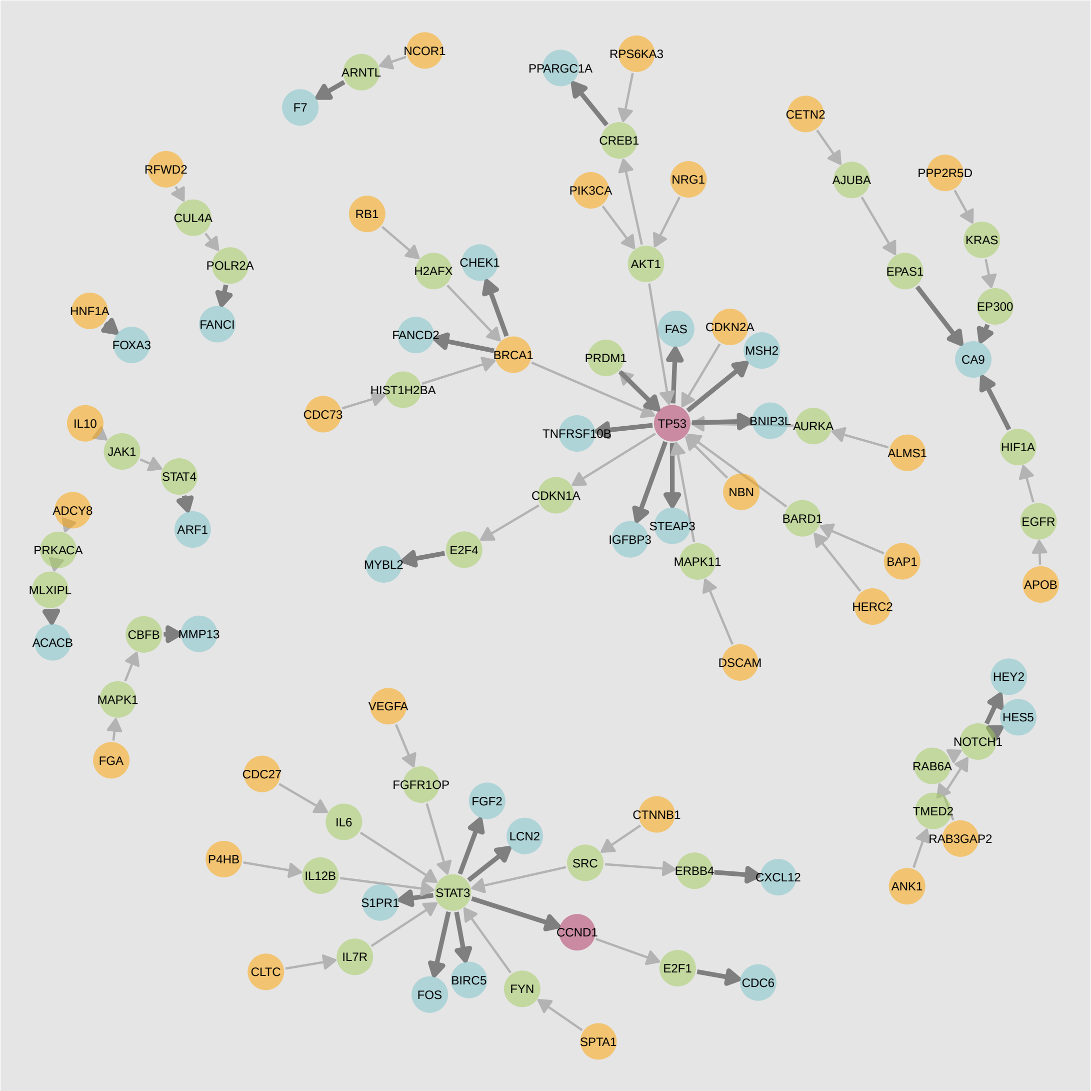

Figure E: Pair covering network for liver cancer. A selected pair covering obtained for liver tumors are presented here as a graphical network; sources are shown in orange, targets in blue, and the intermediary genes in green. Genes that are both source and a targets, are shown in red. Strong connections leading to a target are presented as bolder edges.

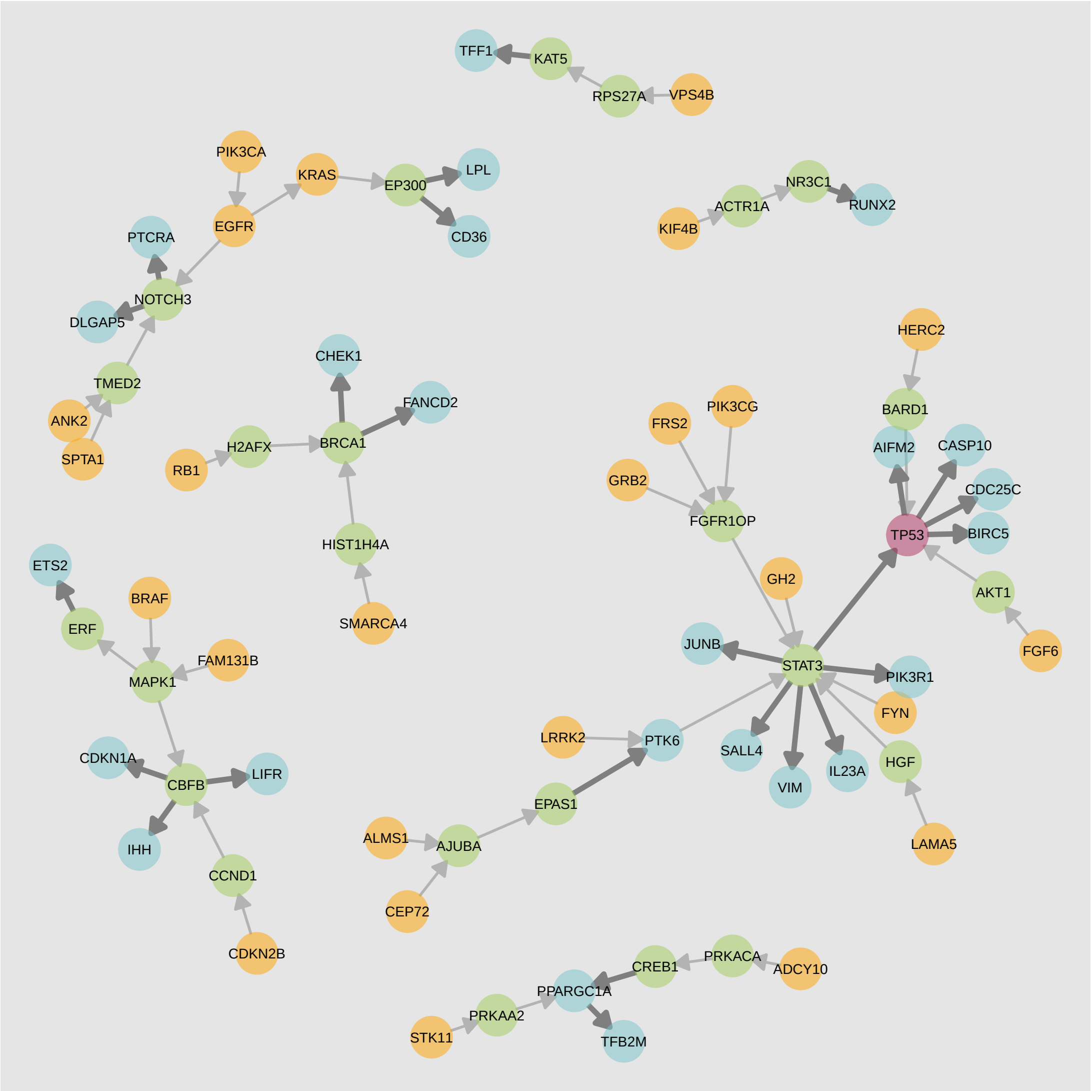

Figure F: Pair covering network for lung cancer. A pair covering obtained for lung tumors is presented as a graphical network. Sources are shown in orange, targets in blue, and the intermediary genes in green. *TP53* which is both source and a target, is shown in red. *TP53, STAT3* and *NOTCH3* form important hubs. Many other known genes of importance such as *KRAS, EGFR* and *MAPK1* can be observed as well. Strong connections are shown in bold edges.

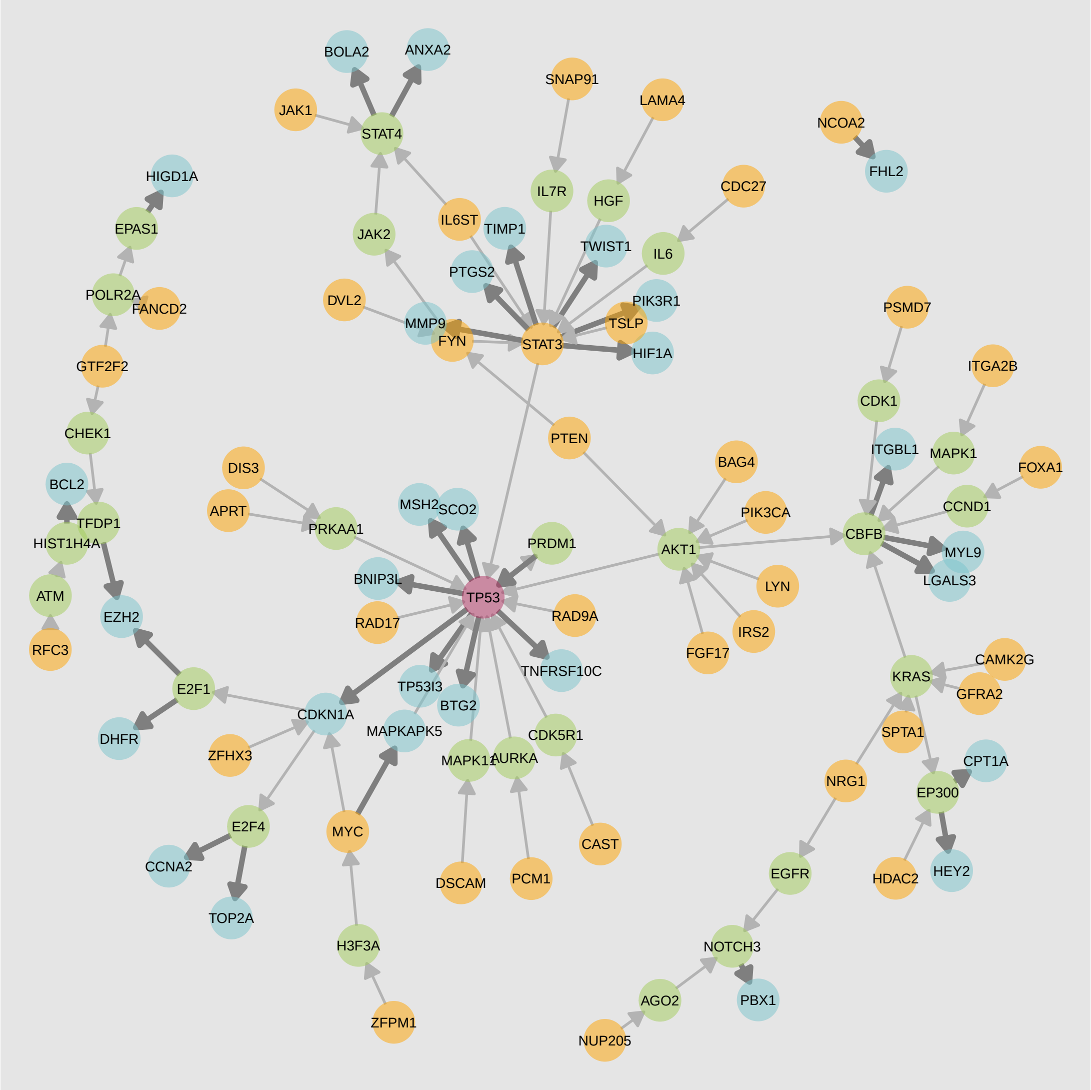

Figure G: Pair covering network for prostate cancer. A selected pair covering obtained for prostate tumors are presented here as a graphical network; sources are shown in orange, targets in blue, and the intermediary genes in green. *TP53* which is both source and a target, is shown in red. Strong connections leading to a target are presented as bolder edges.

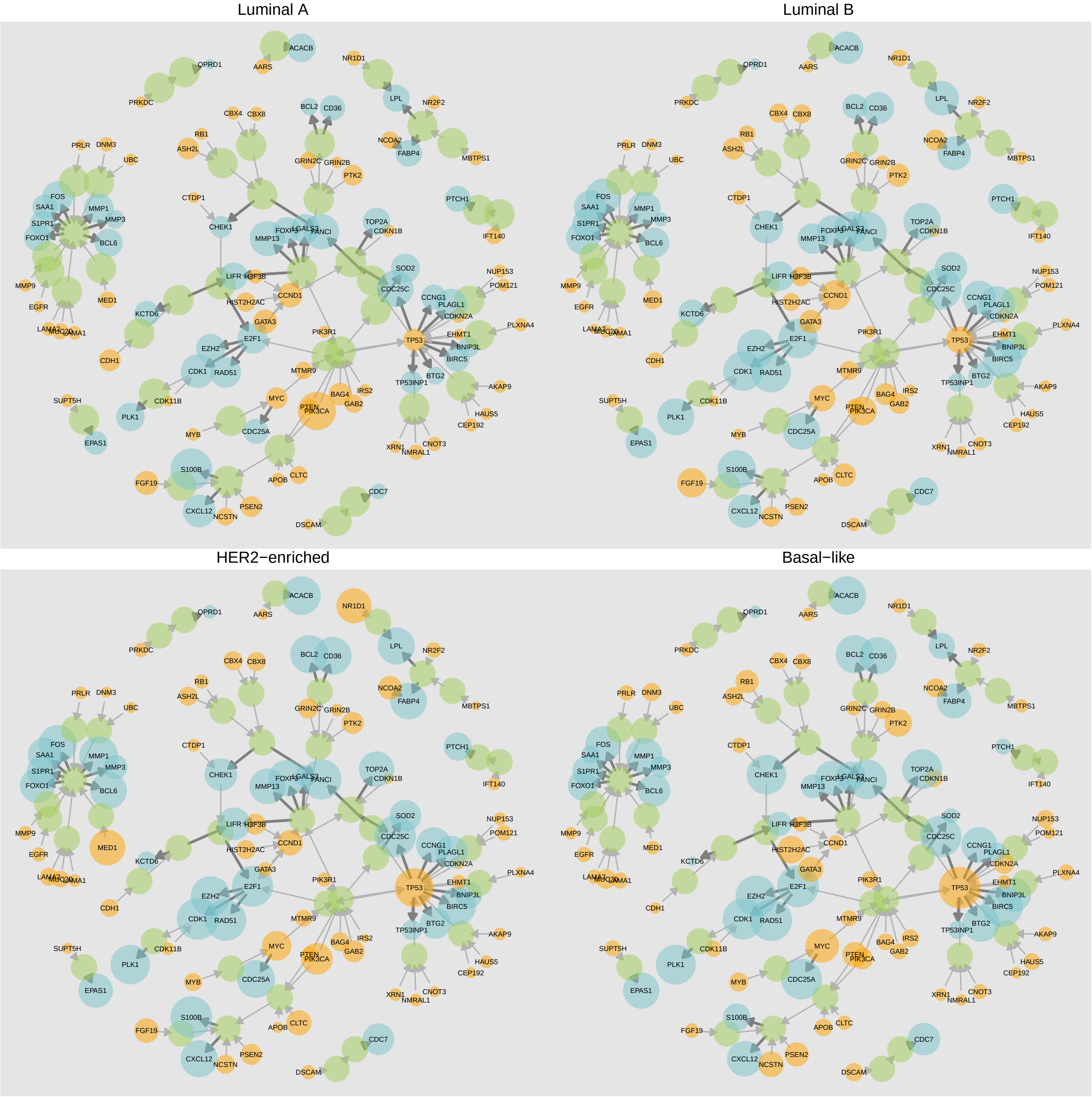

Figure H: Annotated networks for PAM50 breast cancer sub-types. For four PAM50 sub-types considered (with the exception of the Normal-like sub-type) a breast covering presented as a graphical network with the size of nodes scaled to indicate source (with target) aberration probabilities and target (with source) aberration probabilities for samples in the given sub-type.

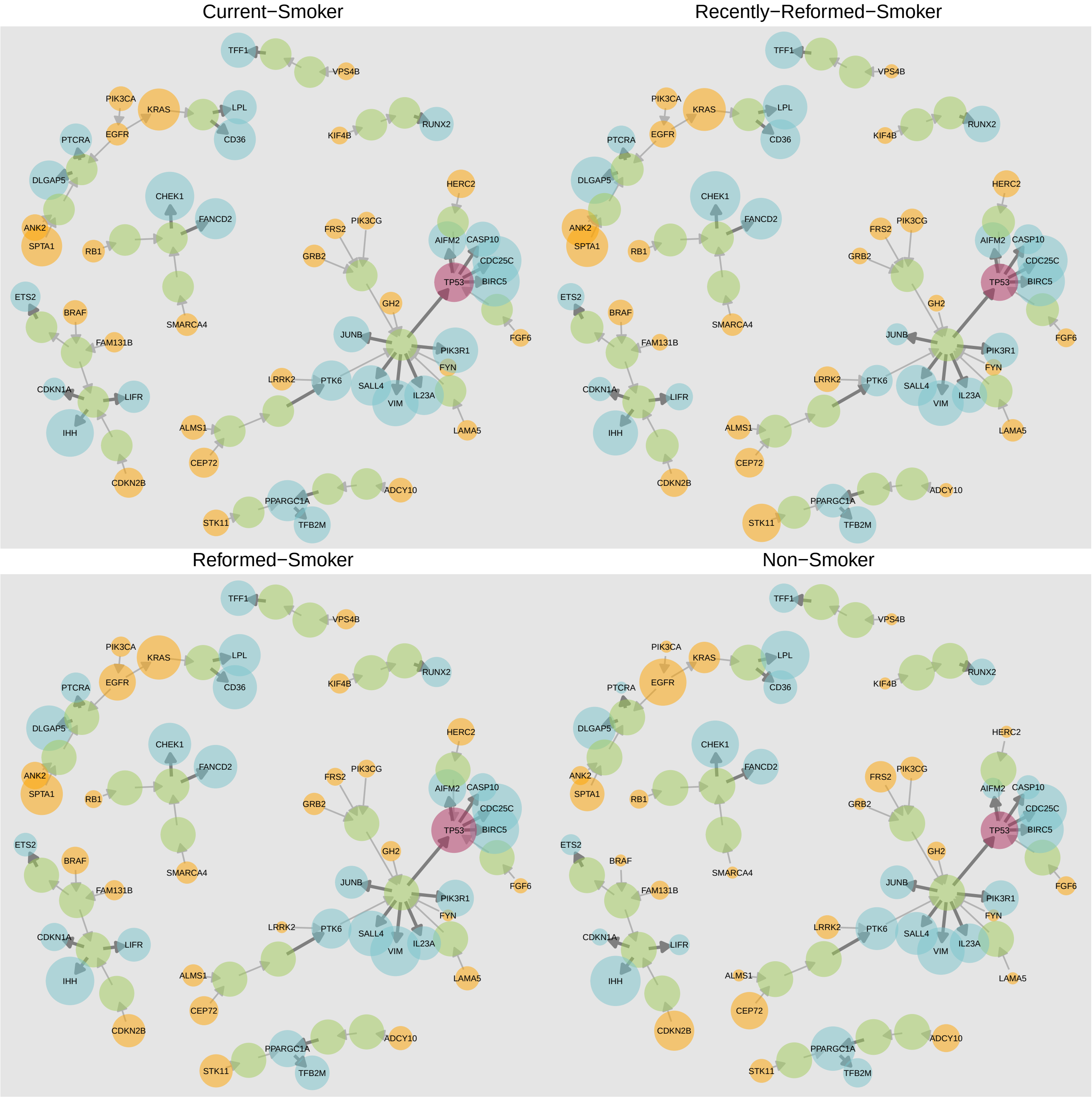

Figure I: Annotated networks for lung cancer based groups based on smoking history. A lung covering is presented here with the size scaled to indicate source (with target) aberration probabilities and target (with source) aberration probabilities for samples in the given sub-type.

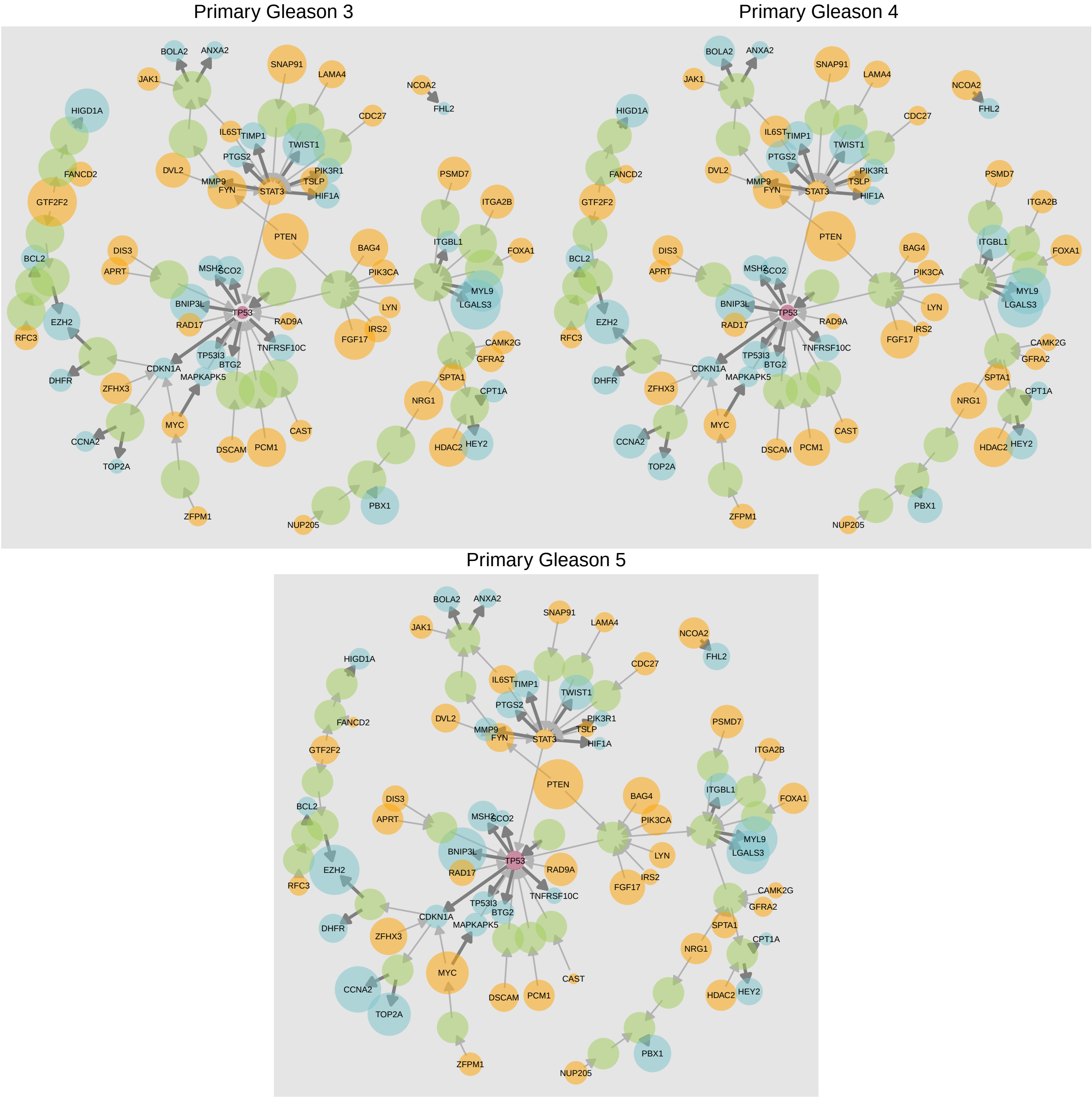

Figure J: Annotated networks for primary Gleason grade in prostate cancer. A prostate covering is presented here with the size scaled to indicate source (with target) aberration probabilities and target (with source) aberration probabilities for samples in the given sub-type.

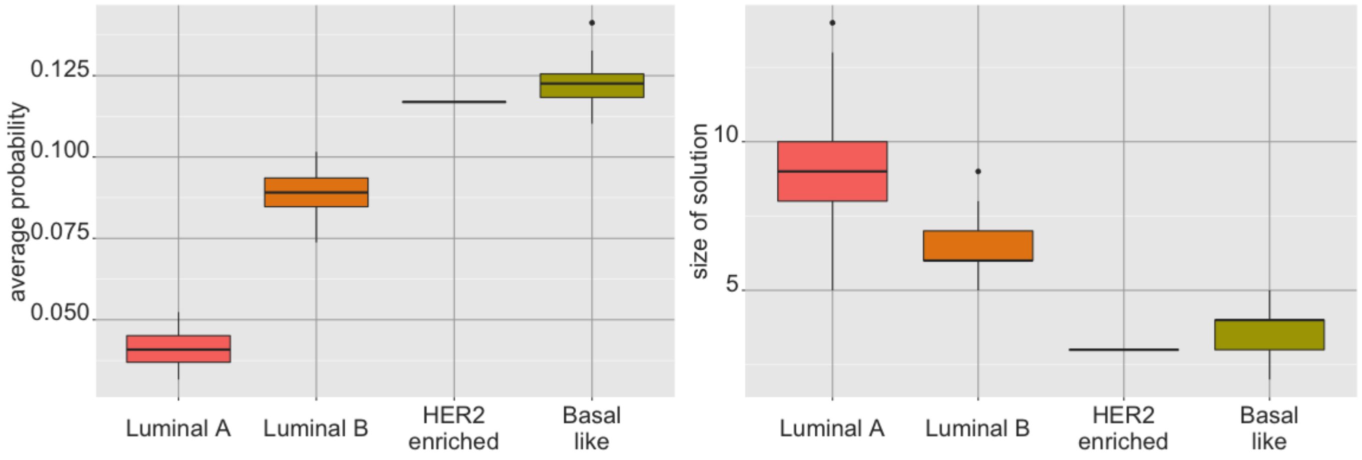

Figure K: Comparison of source sub-type coverings and pair probabilities across breast PAM50 classes. For four PAM50 sub-types considered (with the exception of the Normal-like sub-type), an equal number of samples from each sub-type was randomly sampled. (Left) The average pair aberration probability over the breast pair covering union for 100 iterations of sampling; (Right) The average size of the source covering (computed over the breast source covering union with 90% or more coverage requested for the sub-type) for 100 iterations of such sampling. Between the two Luminal sub-types, the results indicate the more benign Luminal A group exhibiting lower aberration probabilities on average and larger covering sizes than the Luminal B group. A similar pattern is present between the Luminal sub-types and the more malignant Basal-like and HER2-enriched sub-types.

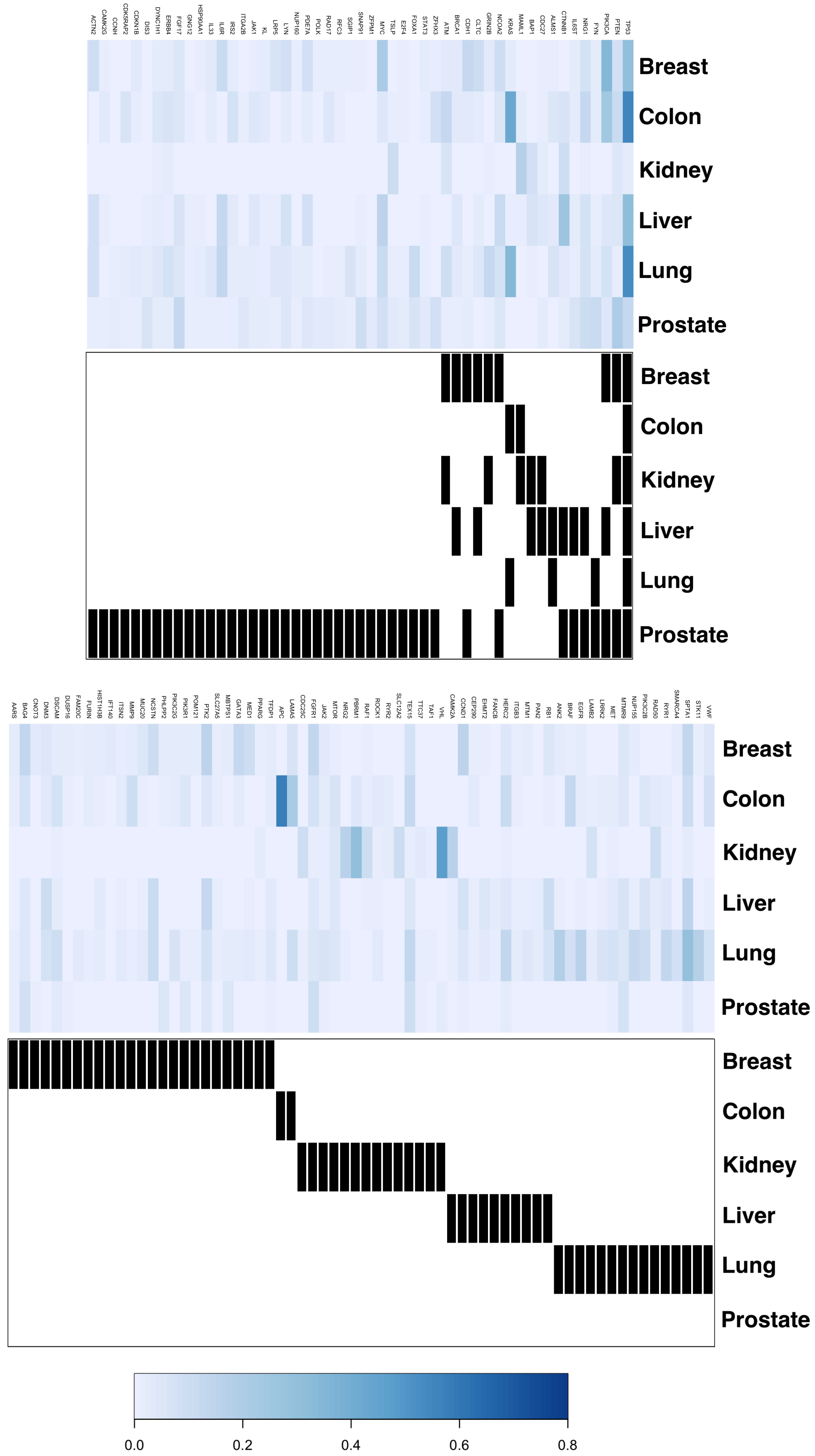

Figure L: Core set across tissues at the source level. Complete core set across tissues at the source level (heatmap split for readability).

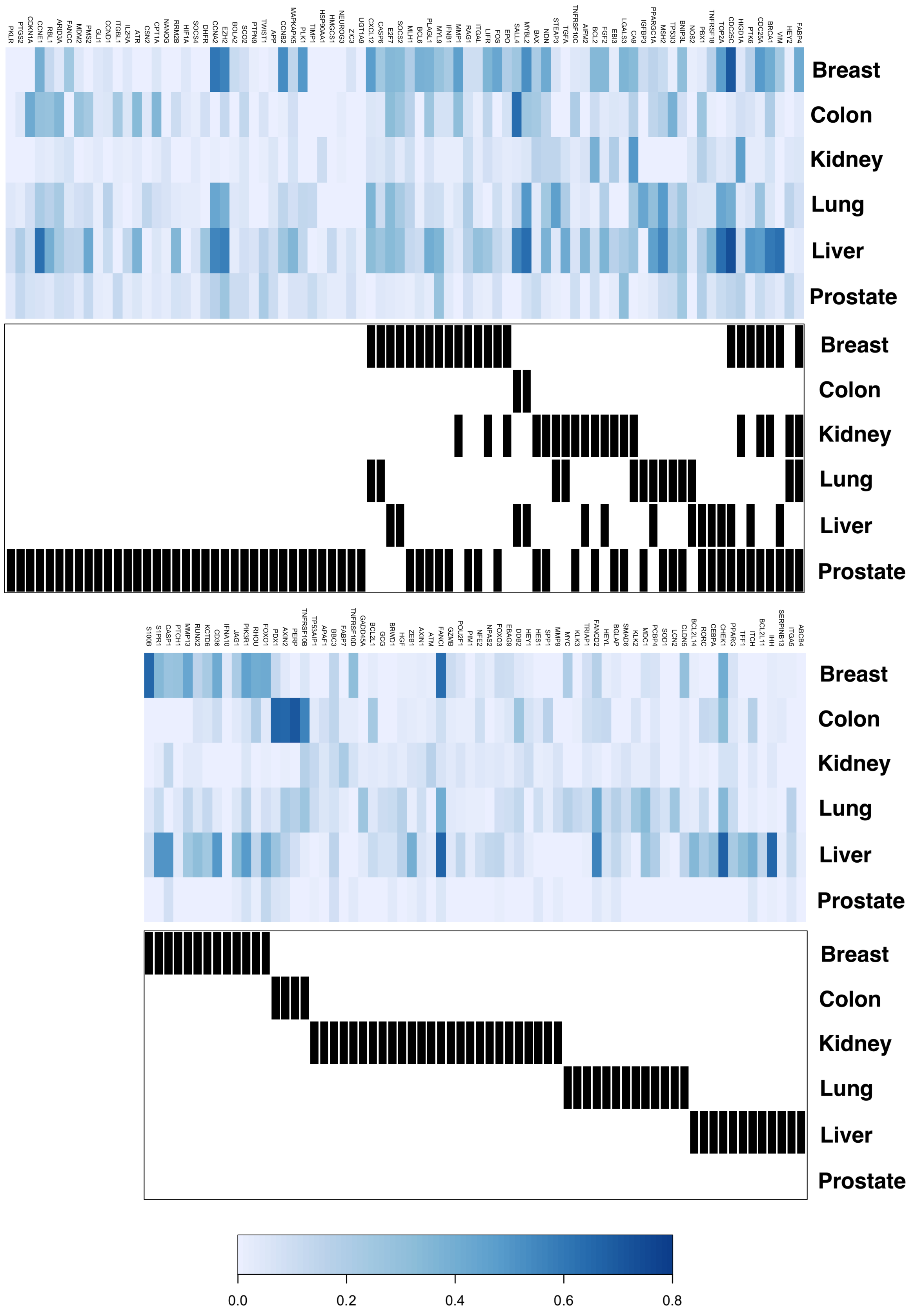

Figure M: Core set across tissues at the target level. Complete core set across tissues at the target level (heatmap split for readability).

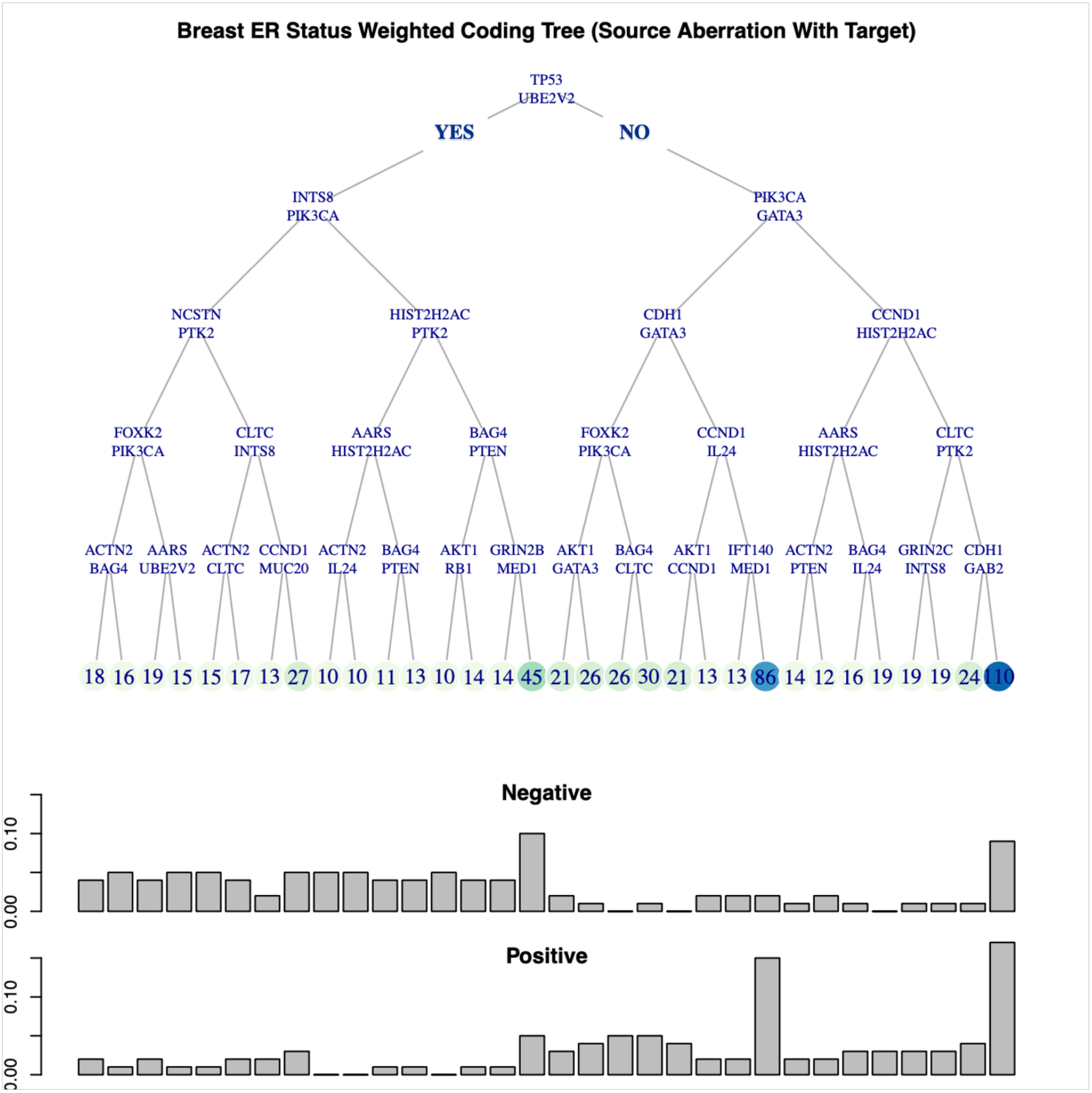

Figure N: Coding tree for breast ER status using source DNA aberration with target. Coding tree for breast ER status using source DNA aberration with target.

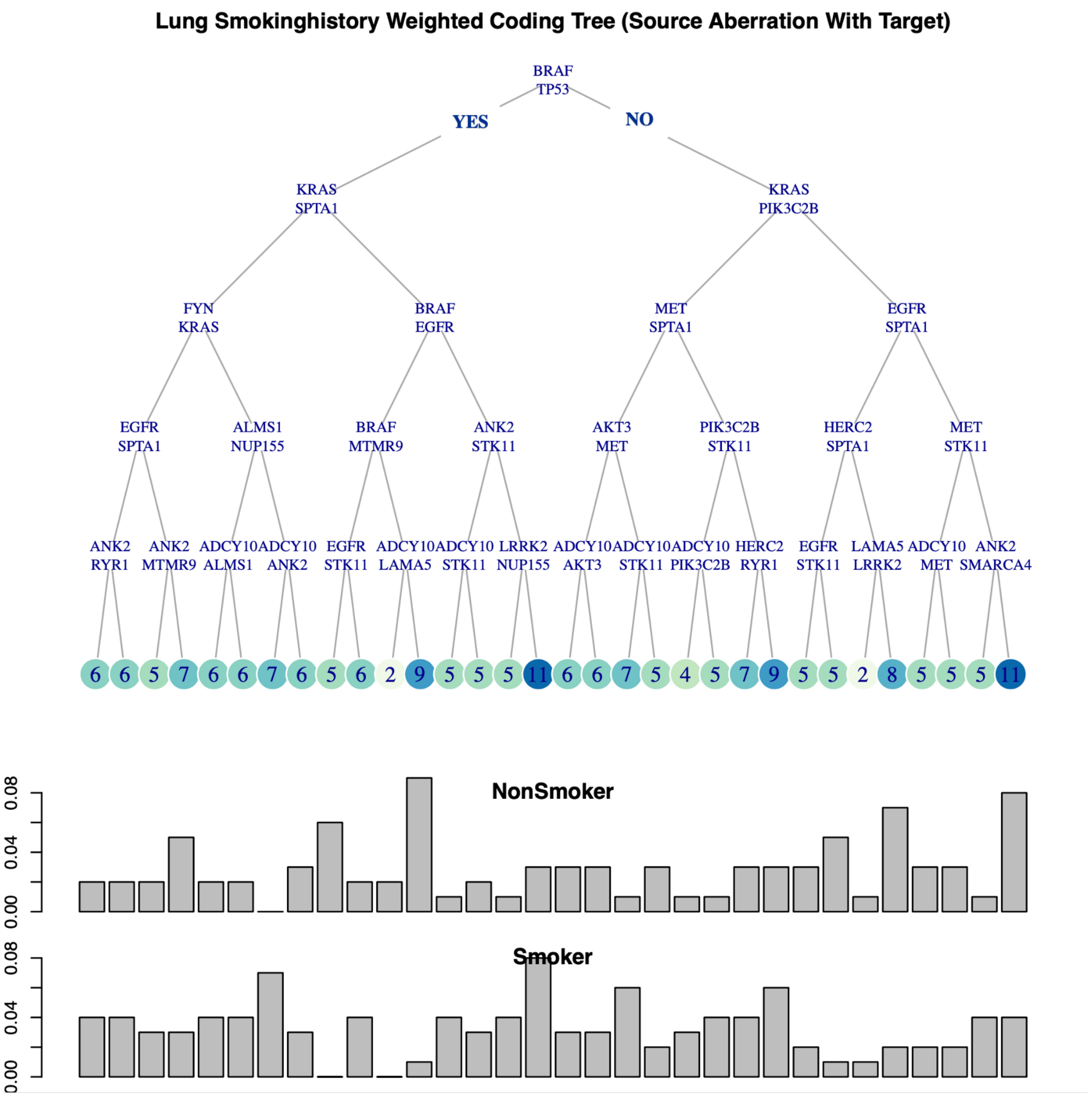

Figure O: Coding tree for lung smoking history using source DNA aberration with target. Coding tree for lung smoking history using source DNA aberration with target.

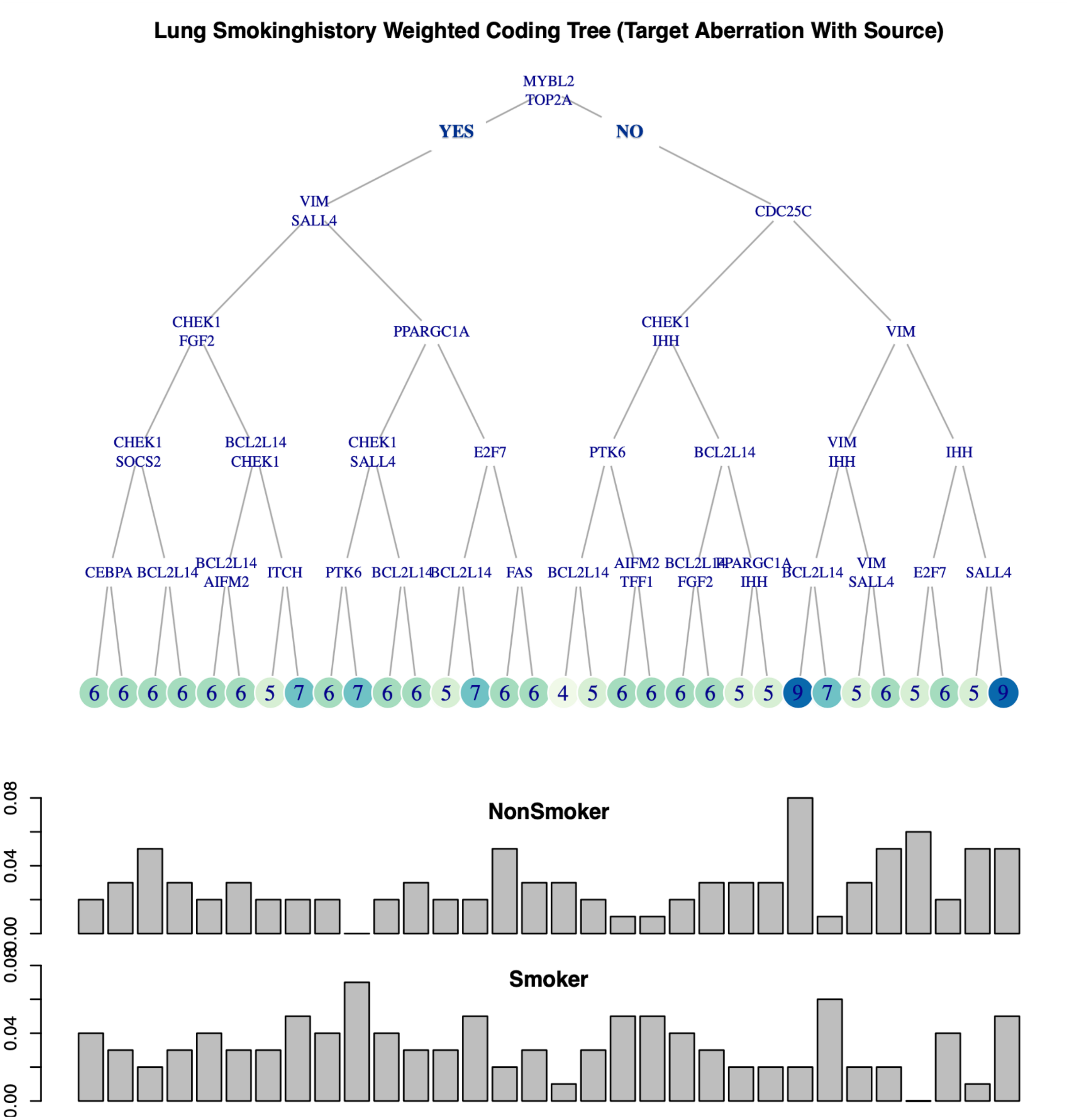

Figure P: Coding tree for lung smoking history using target RNA aberration with source. Coding tree for lung smoking history using target RNA aberration with source.

References

1. Worby CA, Dixon JE. PTEN. Annu Rev Biochem. 2014;83:641–69. doi:10.1146/annurev-biochem-082411-113907.

2. Arafeh R, Samuels Y. PIK3CA in cancer: The past 30 years. Semin Cancer Biol. 2019;59:36–49. doi:10.1016/j.semcancer.2019.02.002.

3. Turner N, Tutt A, Ashworth A. Hallmarks of 'BRCAness' in sporadic cancers. Nat Rev Cancer. 2004;4(10):814–9. doi:10.1038/nrc1457.

4. Eeckhoute J, Keeton EK, Lupien M, Krum SA, Carroll JS, Brown M. Positive cross-regulatory loop ties GATA-3 to estrogen receptor alpha expression in breast cancer. Cancer Res. 2007;67(13):6477–83. doi:10.1158/0008-5472.CAN-07-0746.

5. Goss KH, Groden J. Biology of the adenomatous polyposis coli tumor suppressor. J Clin Oncol. 2000;18(9):1967–79. doi:10.1200/JCO.2000.18.9.1967.

6. Dizman N, Philip EJ, Pal SK. Genomic profiling in renal cell carcinoma. Nat Rev Nephrol. 2020;16(8):435–451. doi:10.1038/s41581-020-0301-x.

7. Oberndorfer F, Müllauer L. Molecular pathology of lung cancer: current status and perspectives. Curr Opin Oncol. 2018;30(2):69–76. doi:10.1097/CCO.0000000000000429.

8. Dong JT. Prevalent mutations in prostate cancer. J Cell Biochem. 2006;97(3):433–47. doi:10.1002/jcb.20696.

9. Arora K, Barbieri CE. Molecular Subtypes of Prostate Cancer. Curr Oncol Rep. 2018;20(8):58. doi:10.1007/s11912-018-0707-9.

10. Cancer Genome Atlas Research Network. The Molecular Taxonomy of Primary Prostate Cancer. Cell. 2015;163(4):1011–25. doi:10.1016/j.cell.2015.10.025.

11. ICGC/TCGA Pan-Cancer Analysis of Whole Genomes Consortium. Pan-cancer analysis of whole genomes. Nature. 2020;578(7793):82–93. doi:10.1038/s41586-020-1969-6.

12. Martincorena I, Raine KM, Gerstung M, Dawson KJ, Haase K, Van Loo P, et al. Universal Patterns of Selection in Cancer and Somatic Tissues. Cell. 2017;171(5):1029–1041.e21. doi:10.1016/j.cell.2017.09.042.

13. Cao H, Gerhold K, Mayers JR, Wiest MM, Watkins SM, Hotamisligil GS. Identification of a lipokine, a lipid hormone linking adipose tissue to systemic metabolism. Cell. 2008;134(6):933–44. doi:10.1016/j.cell.2008.07.048.

14. Varambally S, Dhanasekaran SM, Zhou M, Barrette TR, Kumar-Sinha C, Sanda MG, et al. The polycomb group protein EZH2 is involved in progression of prostate cancer. Nature. 2002;419(6907):624–9. doi:10.1038/nature01075.

15. Charmsaz S, Hughes É, Bane FT, Tibbitts P, McIlroy M, Byrne C, et al. S100β as a serum marker in endocrine resistant breast cancer. BMC Med. 2017;15(1):79. doi:10.1186/s12916-017-0836-2.
